# Supplementary material for: Lysosomal-associated protein transmembrane 5 ameliorates non-alcoholic steatohepatitis by promoting the degradation of CDC42 in mice
Source: Nat Commun. 2023 May 8;14:2654. doi: 10.1038/s41467-023-37908-9 (PMC10167344; doi:10.1038/s41467-023-37908-9)
Supplement: Supplementary file 1 — Supplementary Information [file 41467_2023_37908_MOESM1_ESM.pdf]

Lysosomal-associated protein transmembrane 5 ameliorates non-alcoholic steatohepatitis by promoting the degradation of CDC42 in mice

Lang Jiang[1,6] Jing Zhao[2,6], Qin Yang[3,6], Mei Li[4,6], Hao Liu[1], Xiaoyue Xiao[1], Song Tian[4], Sha Hu[4], Zhen Liu[5], Peiwen Yang[1], Manhua Chen[2*], Ping Ye[2*], Jiahong Xia[1*]

1. Department of Cardiovascular Surgery, Union Hospital, Tongji Medical College, Huazhong University of Science and Technology, Wuhan 430022, China;
2. Department of Cardiology, Central Hospital of Wuhan, Tongji Medical College, Huazhong University of Science and Technology, Wuhan 430014, China;
3. Department of Cardiology, Huanggang Central Hospital, Huanggang, 438021, China;
4. School of Basic Medical Science, Wuhan University, Wuhan, China;
5. Department of Cardiology, Renmin Hospital of Wuhan University, Wuhan, 430060, China;
6. These authors contributed equally to this work.

*Correspondence to: Manhua Chen MD, PHD, Department of Cardiology, Central Hospital of Wuhan, Tongji Medical College, Huazhong University of Science and Technology, ShengLi Street 26, Wuhan 430014, China. Email: cmh_centre@163.com;

Ping Ye MD, PHD, Department of Cardiology, Central Hospital of Wuhan, Tongji Medical College, Huazhong University of Science and Technology, ShengLi Street 26, Wuhan 430014, China. Email: blue314@163.com; Fax: +86-027-82811446;

Jiahong Xia MD, PHD, Department of Cardiovascular Surgery, Union Hospital, Tongji Medical College, Huazhong University of Science and Technology, JieFang Road 1277, Wuhan 430022, China. Email: jiahong.xia@hust.edu.cn; Fax: +86-027-85776364.

Supplementary figures

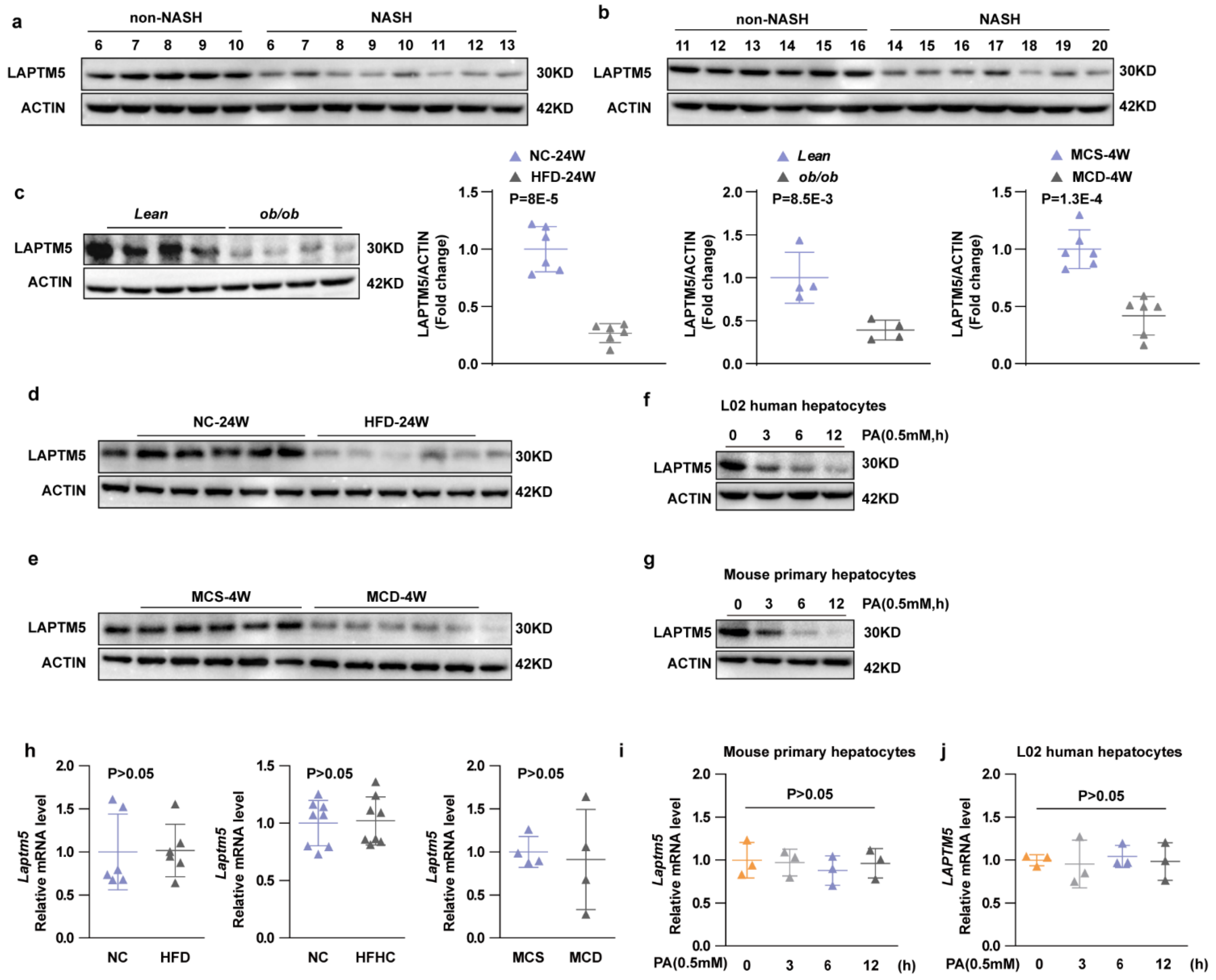

Supplementary Fig. 1. LAPTM5 expression is downregulated in fatty liver.

(a-b) Representative LAPTM5 protein levels in the human livers from NASH (n=20 people) or non-NASH (n=16 people) group. (c-d) Representative the LAPTM5 protein levels in the livers from lean and *ob/ob* mice (n = 4 mice/group) or normal chow diet and HFD fed mice (n = 6 mice/group). (e) LAPTM5 protein levels in the livers from MCS and MCD fed mice (n = 6 mice/group). (f-g) Representative LAPTM5 protein levels in PA-stimulated L02 human hepatocytes (f) and primary hepatocytes (g), (n = 3 independent experiments). (h) Relative *Laptm5* mRNA levels in the mice livers from the indicated NAFLD or NASH models (left, n =

6 mice/group, middle, n = 8 mice/group, right, n = 4 mice/group). (i-j) Relative *Laptm5* mRNA levels in primary hepatocytes (i) and L02 hepatocytes (j) stimulated with PA (0.5 mM) (n = 3 independent experiments). For (c-e), Protein expression was normalized to β-ACTIN. Data are presented as mean ± SD, n.s., not significant. The two-tailed Student's t-test was used for statistical analysis in (c-e,and h-middle and right), and Mann-Whitney U nonparametric statistical test in (h-left). One-way ANOVA with Bonferroni's post hoc analysis for (i) and Tamhane T2 post-hoc test for (j). Source data are provided as a Source data file.

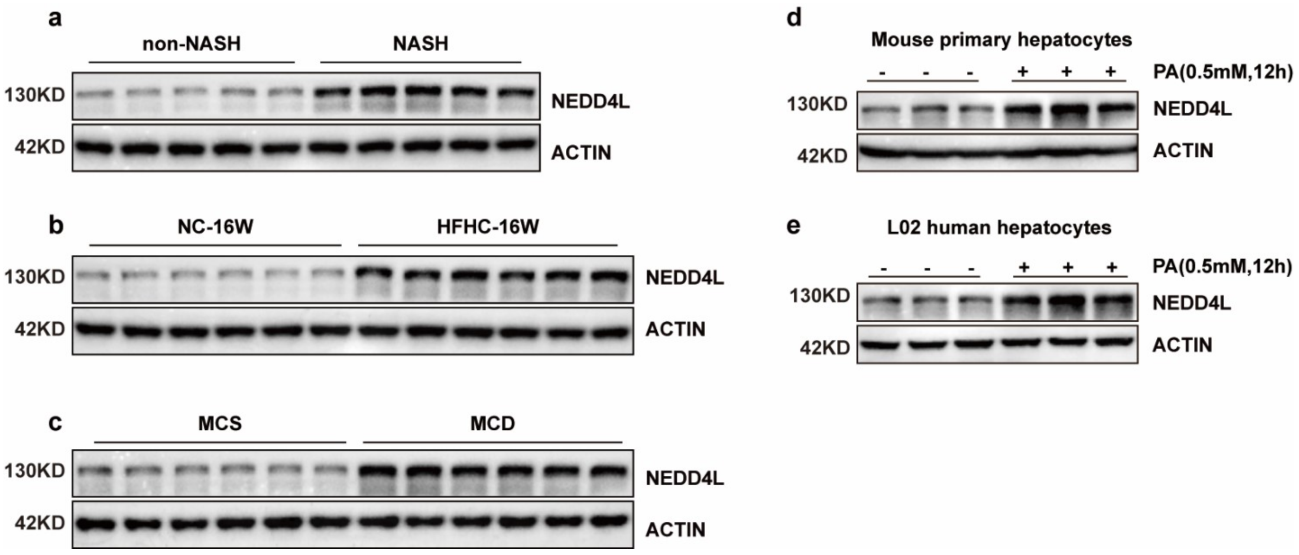

Supplementary Fig. 2. NEDD4L is regulated by NASH.

(a) Representative NEDD4L protein level in the human livers from NASH or non-NASH group (n = 5 people/group). (b-c) Representative the NEDD4L protein levels in the livers from normal chow diet and HFHC diet fed mice (n = 6 mice/group) or MCS and MCD fed mice (n = 6 mice/group). (d-e) Representative NEDD4L protein levels in PA-stimulated primary hepatocytes (d) and L02 human hepatocytes (e), (n = 3 independent experiments). Source data are provided as a Source data file.

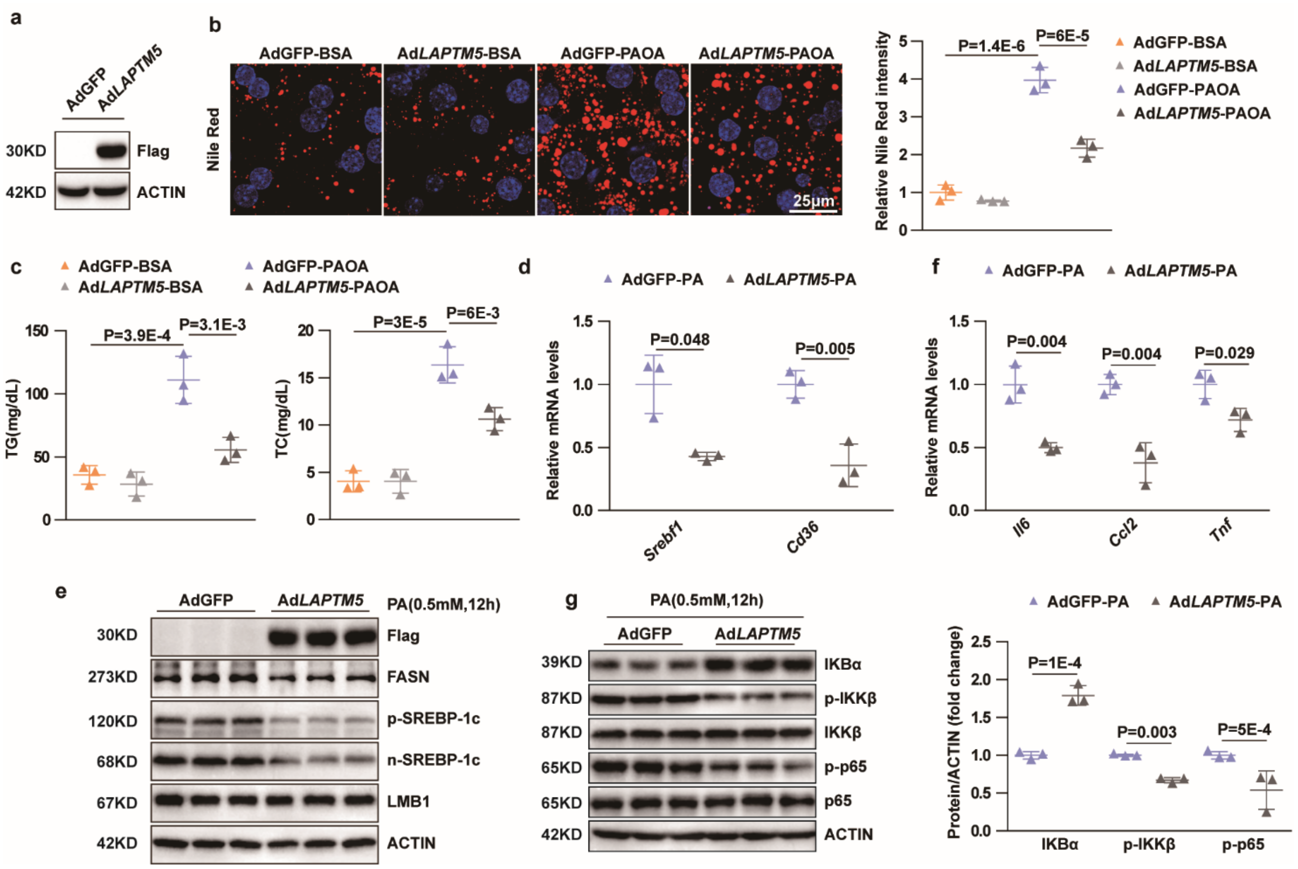

Supplementary Fig. 3. LAPTM5 inhibits lipid accumulation and inflammation in hepatocytes.

(a) Represents LAPTM5 protein levels in mouse primary hepatocytes in the indicated groups. (n = 3 independent experiments). (b-c) Nile Red staining (b) and TG, TC contents (c) in mouse primary hepatocytes in the indicated groups. Scale bar, 25μm.(n = 3 independent experiments). (d-e) Relative mRNA (d) and protein (e) levels of markers related to fatty acid metabolism in the indicated groups. (n = 3 mice/group). (f-g) Relative mRNA (f) and protein (g) levels of markers related to inflammation in the indicated groups. Protein expression was normalized to β-ACTIN.(n = 3 mice/group). PAOA, 0.5 mM/1.0 mM; PA, 0.5 Mm, OA, 1.0mM. Data are represented as mean ± SD. Data were subjected to one-way ANOVA of Bonferroni post-hoc test for (b-c), and two-tailed Student's t-test for (d, f, g). Source data are provided as a Source data file.

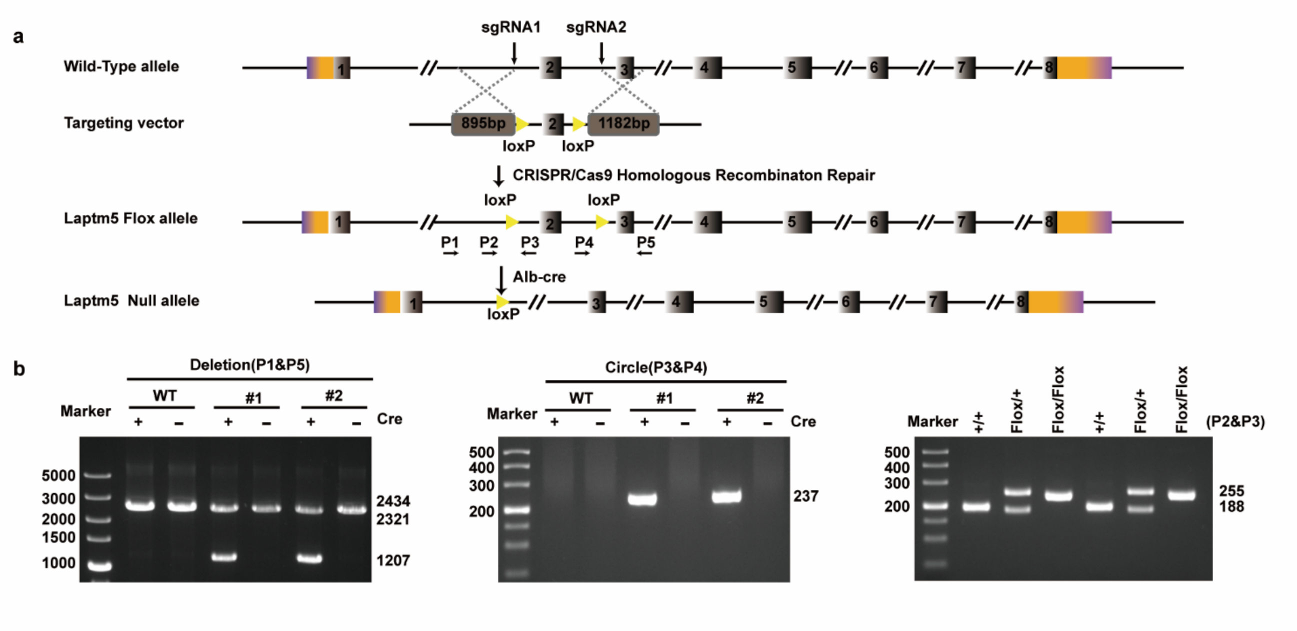

Supplementary Fig. 4. Rudimentary diagram of the generation of *Laptm5* hepatocyte-specific knockout mice.

(a-b) Rudimentary diagram of the generation of *Laptm5* hepatocyte-specific knockout mice *Laptm5*-HKO (a) and genotyping results (b).

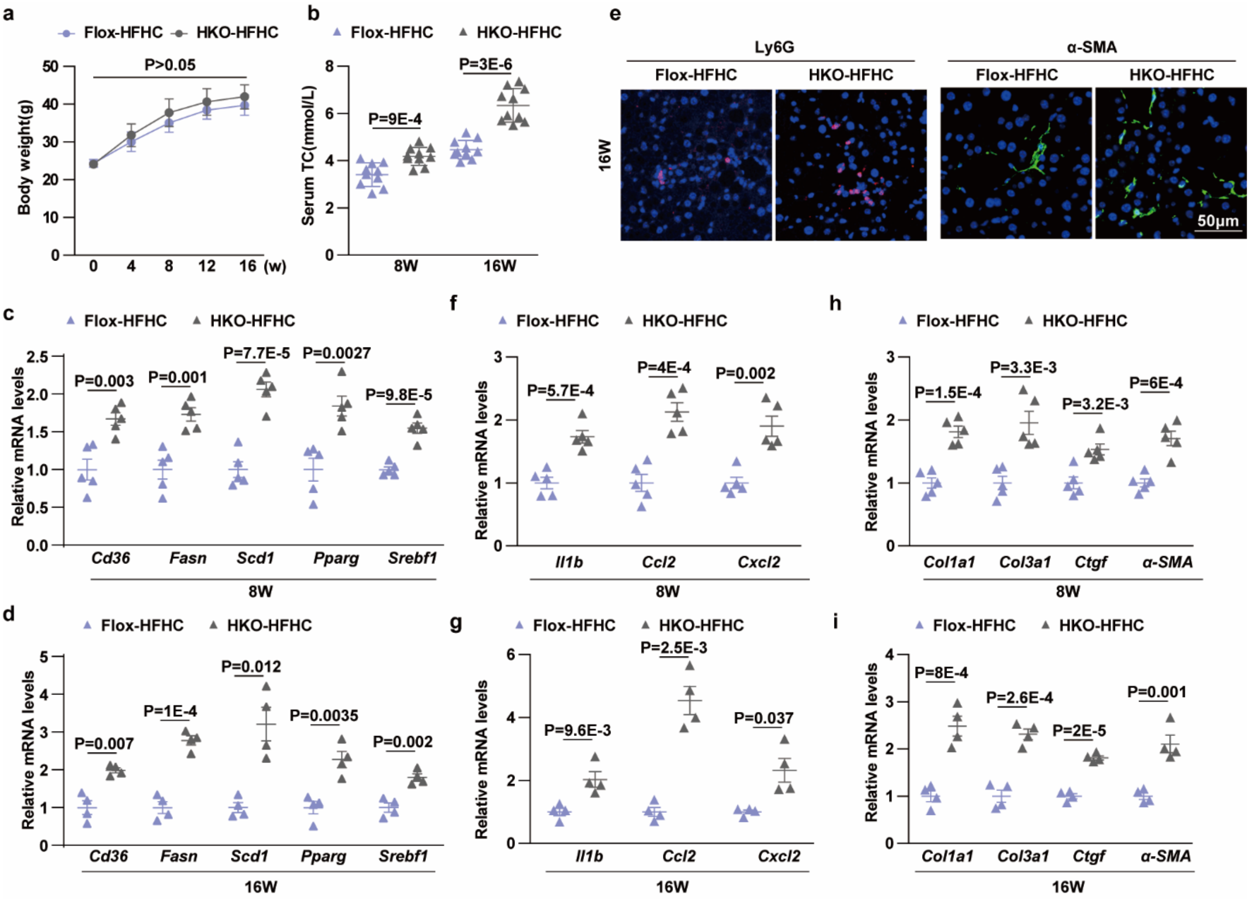

Supplementary Fig. 5. *Laptm5*-HKO exacerbates HFHC-induced NASH.

(a) Body weights of *Laptm5*-HKO and *Laptm5*-Flox mice for HFHC consumptions every 4 weeks until 16 weeks (n = 10 mice/group). (b) serum TC of mice after HFHC diet for 8 or 16 weeks in the indicated groups (n = 10 mice/group). (c-d) Relative mRNA levels of lipid metabolism related genes in the livers of mice in the indicated groups (n = 4 mice/group). (e) Immunofluorescence staining of Ly6G (red) (n = 4 mice/group) and α-SMA (n = 6 mice/group) in the liver sections of mice in the indicated groups. (Nuclei, blue). Scale bar, 50μm. (f-g) Relative mRNA levels of pro-inflammatory cytokines in the livers of mice in the indicated groups (n = 4 mice/group). (h-i) Relative mRNA levels of fibrosis related genes in the livers of mice in the indicated groups (n = 4 mice/group). Data are represented as mean ± SD, two-tailed Student's t-test was used to evaluate differences in all panels. Source data are provided

as a Source data file.

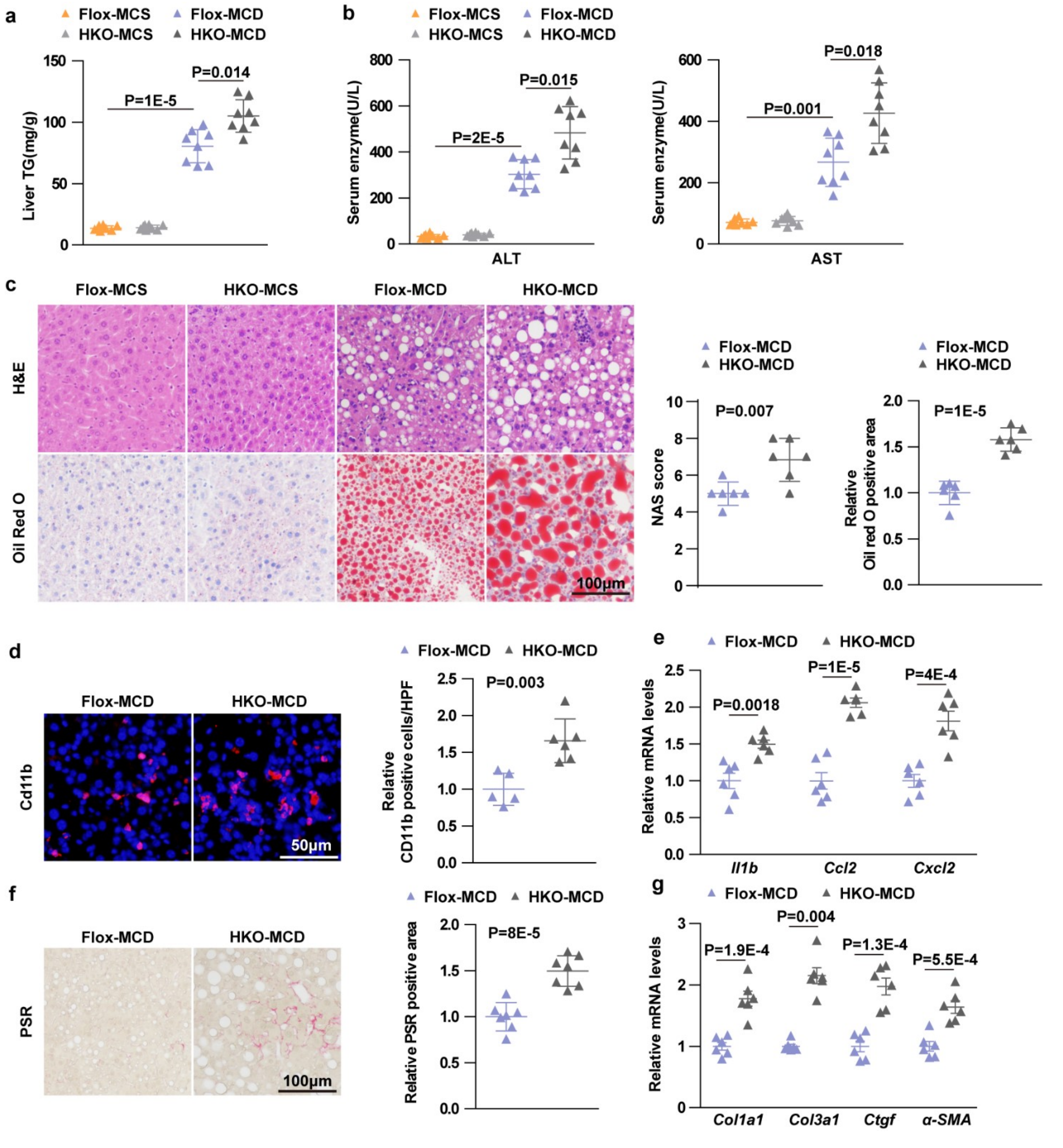

Supplementary Fig. 6. *Laptm5*-HKO exacerbates MCD-induced NASH.

(a-b) Hepatic TG contents (a), serum ALT and AST concentrations (b) of *Laptm5*-HKO and *Laptm5*-Flox mice after MCS or MCD consumptions for 4 weeks (n = 8 mice/group). (c) H&E (upper) and Oil Red O (lower) staining in the liver sections of *Laptm5*-HKO and *Laptm5*-Flox

mice after MCS or MCD consumption for 4 weeks (n = 6 mice/group). Scale bar, 100μm. (d) Immunofluorescence staining of CD11b (red) in the liver sections of mice in the indicated groups. Nuclei in immunofluorescence images were stained with DAPI (blue) (n = 5 *Laptm5*-Flox mice and n=6 *Laptm5*-HKO mice in MCD group). Scale bars, 50μm. (e) Relative mRNA levels of genes in livers of mice in the indicated groups (n = 6 mice/group). (f) PSR staining in the liver sections of mice in the indicated groups (n =7 mice/group). Scale bar, 100μm. (g) Relative mRNA levels of genes in livers of mice in the indicated groups (n = 6 mice/group). Data are represented as mean ± SD. One-way ANOVA of Tamhane T2 post-hoc test was used to evaluate differences in (a-b) and two-tailed Student's t-test in (c-g). Source data are provided as a Source data file.

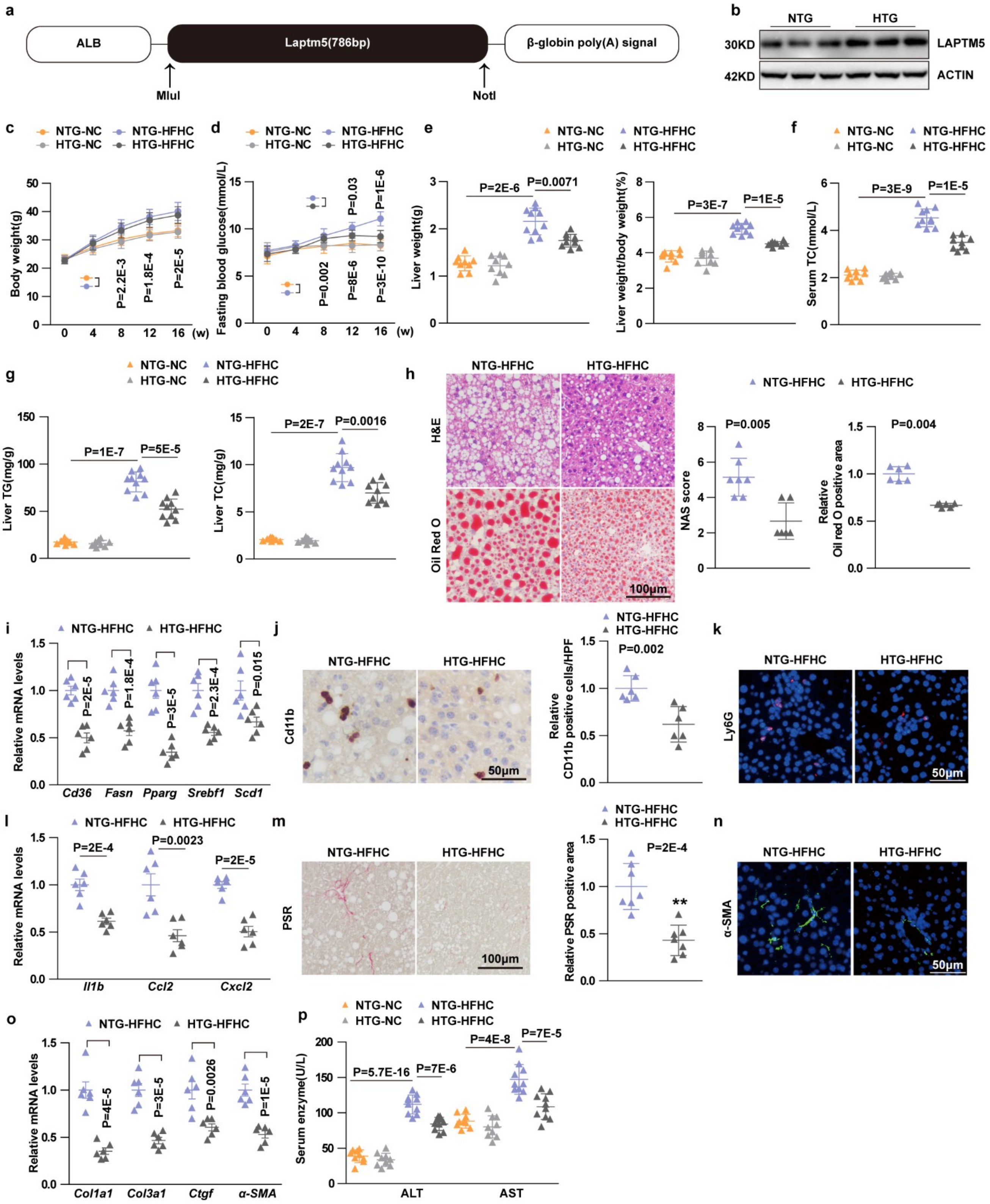

Supplementary Fig. 7. Hepatocyte-specific *Laptm5* overexpression mitigates HFHC-induced NASH.

(a) Rudimentary diagram of the generation of hepatocyte-specific *Laptm5* transgenic

(*Laptm5*-HTG) mice. (b) Western blot images of LAPTM5 protein levels from liver tissues of *Laptm5*-HTG or *Laptm5*-NTG mice (n = 3 mice/group). (c-e) Body weight (c), fasting blood glucose (d), liver weight and LW/BW (e) of *Laptm5*-HTG and *Laptm5*-NTG mice after NC or HFHC consumptions for 16 weeks. (f-g) Serum TC (f), hepatic TG and TC contents (g) of mice in the indicated groups. For (c-g), n=9 mice/NC group and n=10 mice/HFHC group. (h) H&E (upper) and Oil Red O (lower) staining of mice liver sections in the indicated groups. For H&E,n=7 NTG mice and n=6 HTG mice/HCHC group, For Oil red O,n = 6 mice/group. Scale bar, 100μm. (i) Relative mRNA levels of lipid metabolism related genes in the livers of mice in the indicated groups (n = 6 mice/group). (j-k) Immunohistochemistry staining of CD11b (j) (n = 6 mice/group) and immunofluorescence staining of Ly6G (k) (n = 4 mice/group) in liver sections of mice in the indicated groups. Scale bar, 50μm. (l) Relative mRNA levels of pro-inflammatory genes in livers of mice in the indicated groups (n = 6 mice/group). (m-n) PSR staining (m) (n = 7 mice/group) and α-SMA staining (n) (n = 6 mice/group) in liver sections of mice in the indicated groups.Scale bar, 100μm (m), 50μm (n). (o) Relative mRNA levels of profibrotic genes in livers of mice in the indicated groups (n = 6 mice/group). (p) Serum ALT and AST concentrations of mice in the indicated groups.(n=9 mice/NC group and n=10 mice/HFHC group). Data are represented as mean ± SD, one-way ANOVA of Bonferroni post-hoc test (c, d and p) and Tamhane T2 post-hoc test (e-g) was used to evaluate differences.The two-tailed Student's t-test (i-m, and o) and Mann-Whitney U nonparametric statistical test (h) were used to evaluate differences. Source data are provided as a Source data file.

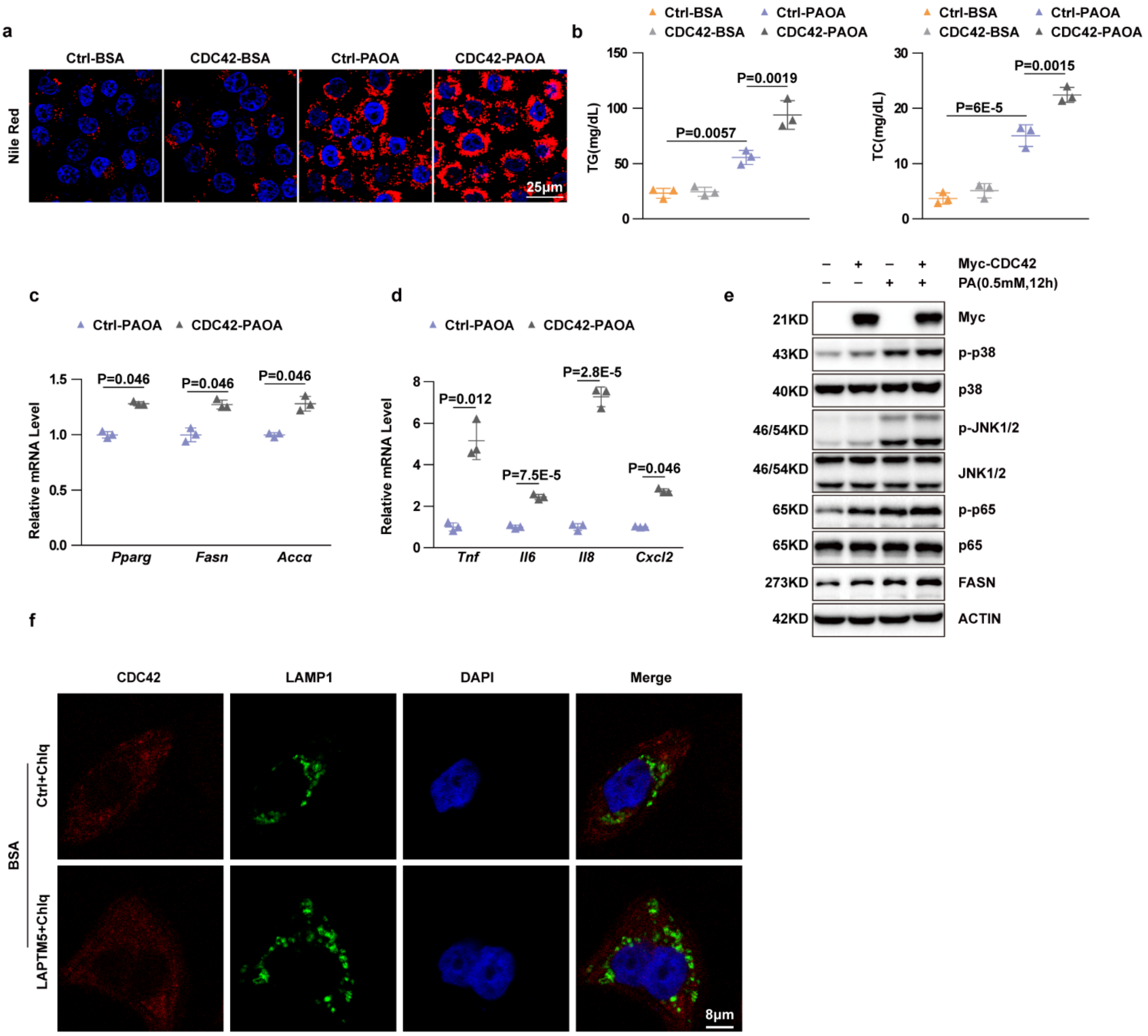

Supplementary Fig. 8. Hepatocyts overexpression of CDC42 aggravates NASH.

(a-b) Nile Red staining (a) and TG, TC contents (b) of L02 cells in the indicated groups. Scale bar, 25μm.(n=3 independent experiments). (c-d) Relative mRNA levels of lipid metabolism (c) and inflammation (d) related genes in the indicated groups.(n=3 independent experiments). (e) Western blot results of the expression of MAPK signaling pathway, lipid metabolism and inflammation-related proteins.(n=3 independent experiments). (f) Confocal microscopy images of the co-localization of LAMP1 (green) and CDC42 (red) in L02 cells in the indicated groups (Nuclei, blue). Scale bar, 8μm.(n=3 independent experiments). PAOA, 0.5 mM/1.0

mM; PA, 0.5 mM, OA, 1.0 mM. Data are represented as mean ± SD. One-way ANOVA of Bonferroni post-hoc test was used to evaluate differences in (b).The two-tailed Student's t-test (d-*Tnf*, *Il6* and *Il8*) and Mann-Whitney U nonparametric statistical test (c and d-*Cxcl2*) were used to evaluate differences. Source data are provided as a Source data file.

Supplemental materials

Uncropped gels for Western blot

Uncropped gels for Western blot in figure 1

g

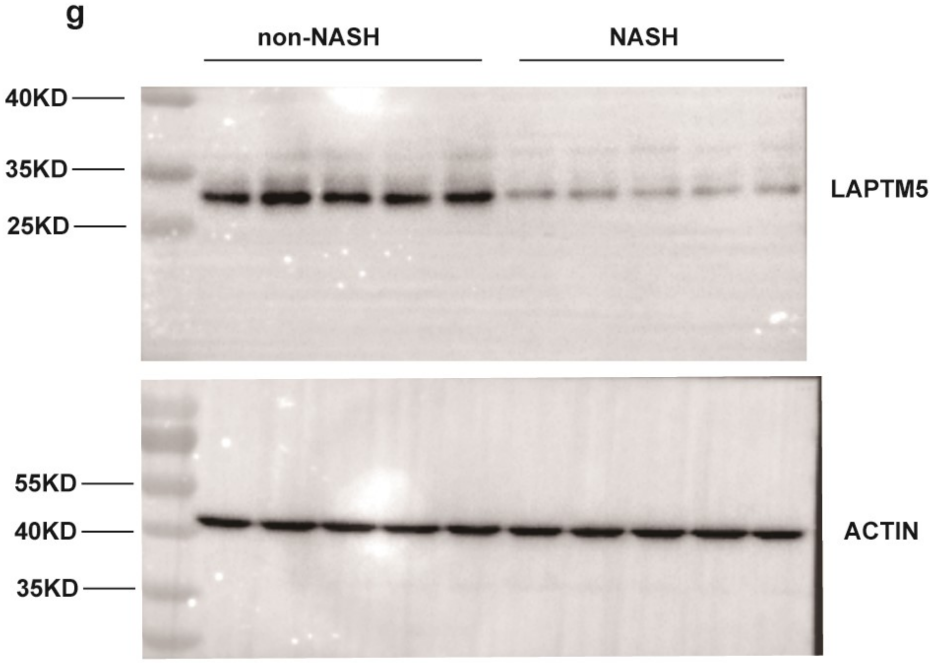

j

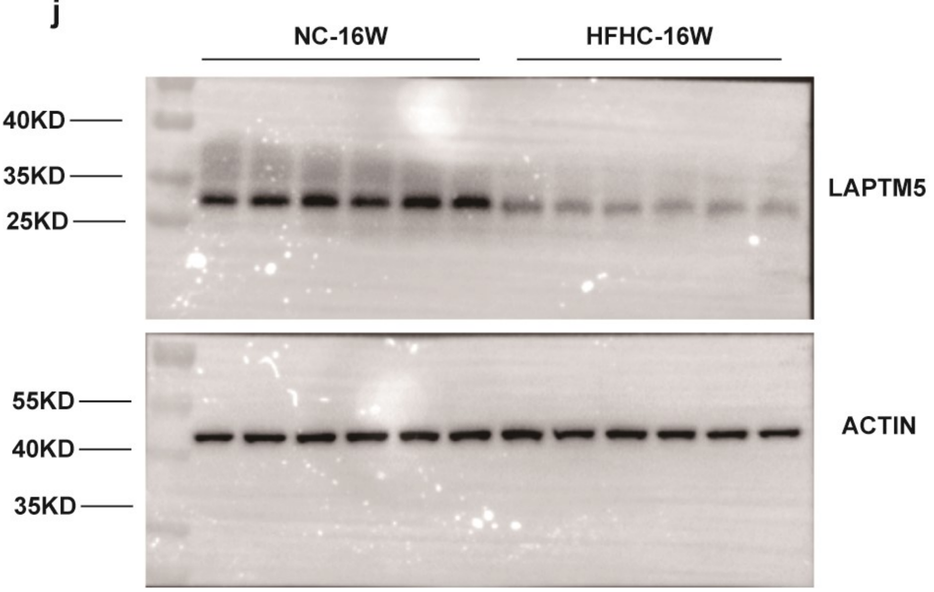

Uncropped gels for Western blot in figure 2

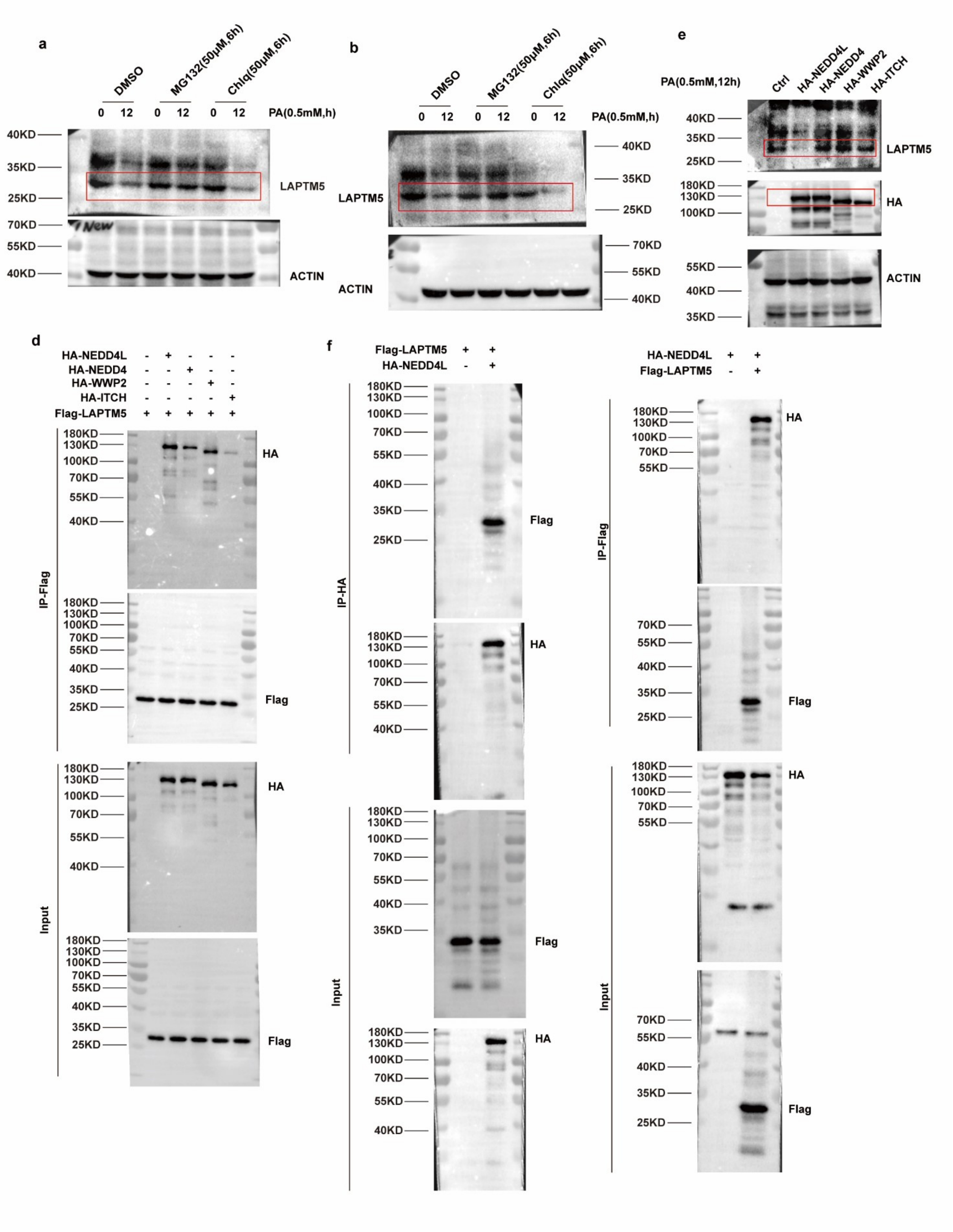

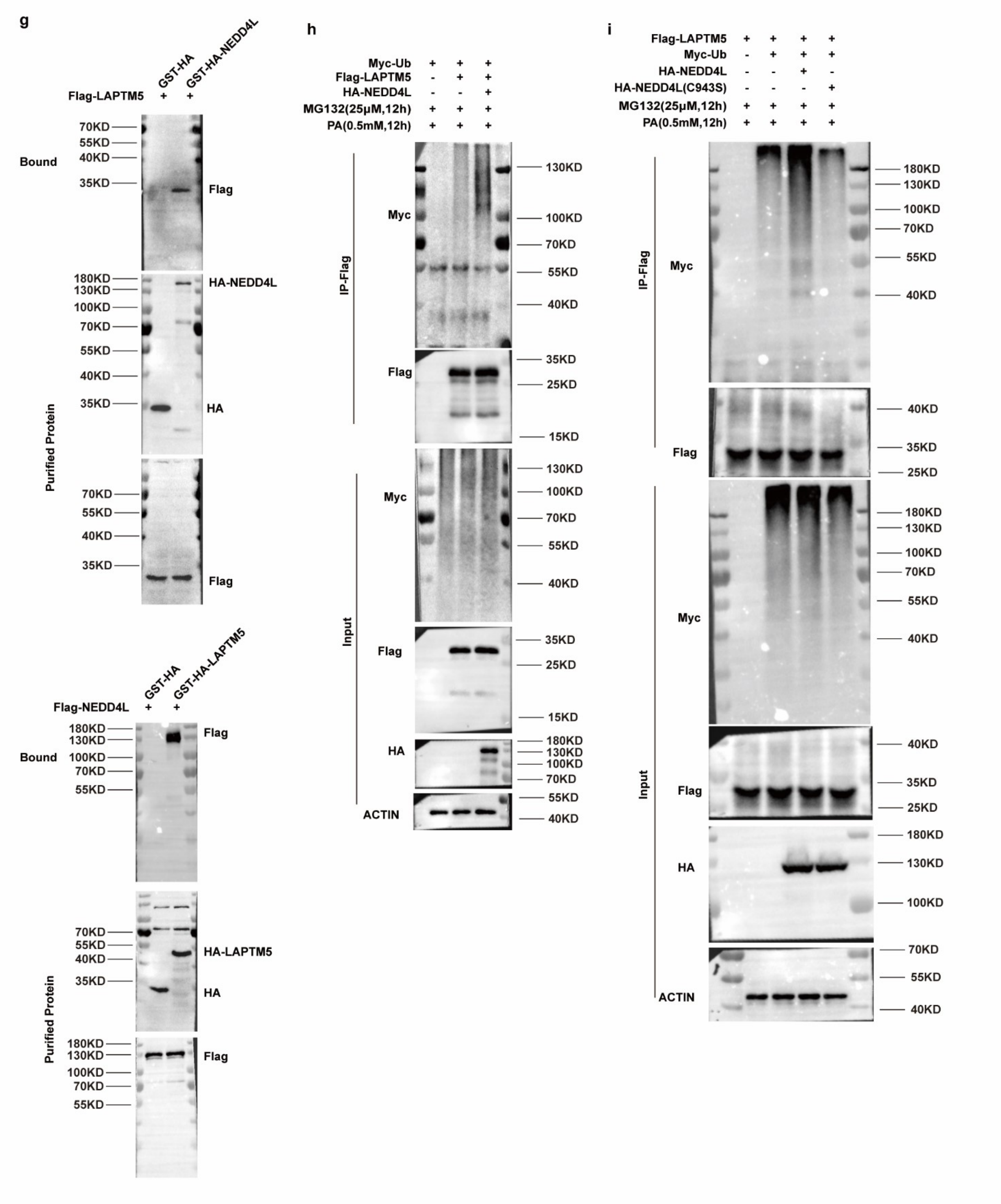
g
GST-HA
GST-HA-NEDD4L
Flag-LAPTM5
Bound
Flag
HA-NEDD4L
HA
Purified Protein
Flag
GST-HA
GST-HA-LAPTM5
Flag-NEDD4L
HA-LAPTM5
h
Myc-Ub
Flag-LAPTM5
HA-NEDD4L
MG132(25μM,12h)
PA(0.5mM,12h)
IP-Flag
Myc
Input
ACTIN
i
HA-NEDD4L(C943S)

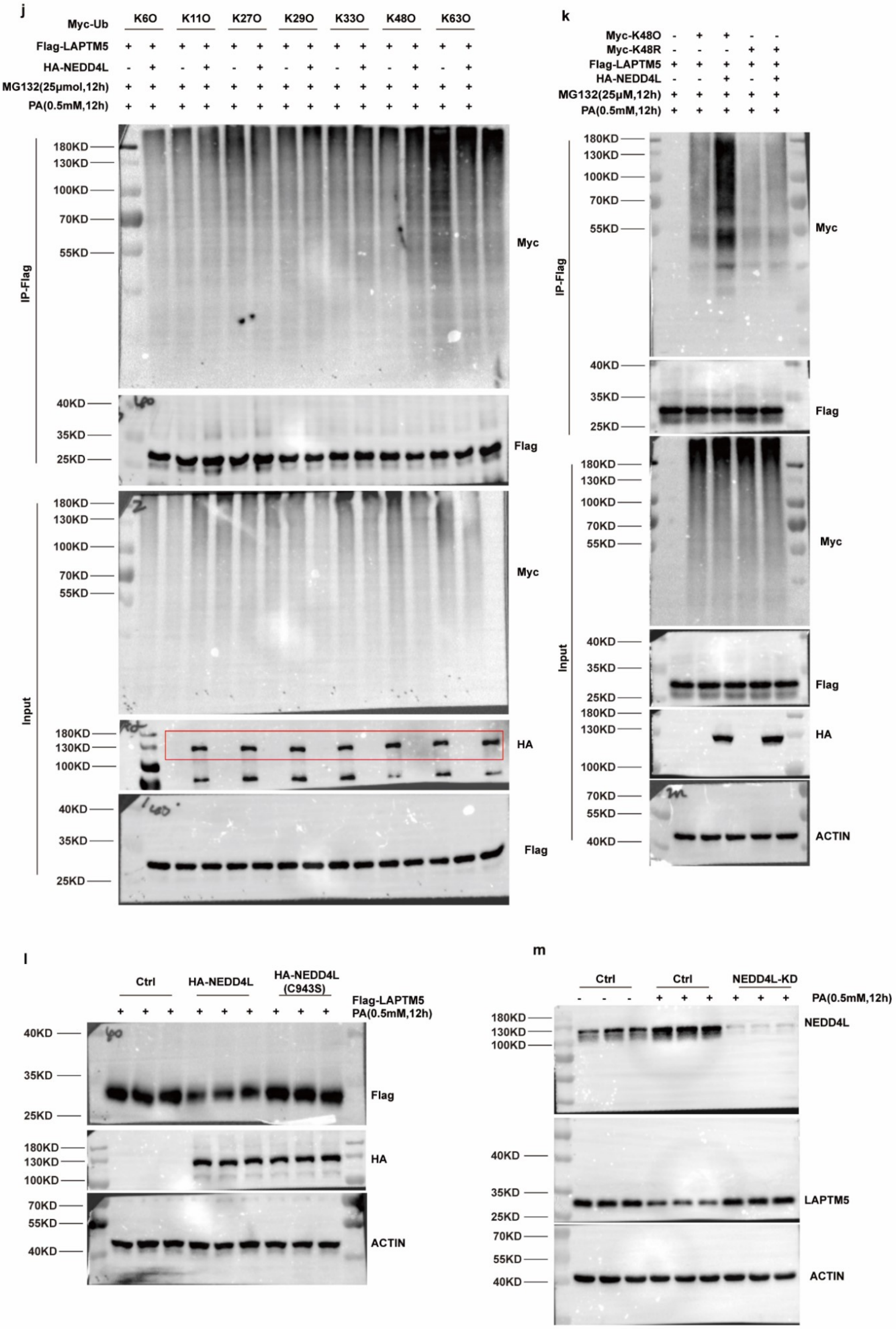
j
Myc-Ub
K6O
K11O
K27O
K29O
K33O
K48O
K63O
Flag-LAPTM5
HA-NEDD4L
MG132(25μmol,12h)
PA(0.5mM,12h)
IP-Flag
Input
180KD
130KD
100KD
70KD
55KD
40KD
35KD
25KD
Myc
Flag
HA
k
Myc-K48O
Myc-K48R
Flag-LAPTM5
HA-NEDD4L
MG132(25μM,12h)
PA(0.5mM,12h)
ACTIN
l
Ctrl
HA-NEDD4L
HA-NEDD4L (C943S)
Flag-LAPTM5
PA(0.5mM,12h)
m
Ctrl
NEDD4L-KD
NEDD4L
LAPTM5

Uncropped gels for Western blot in figure 3

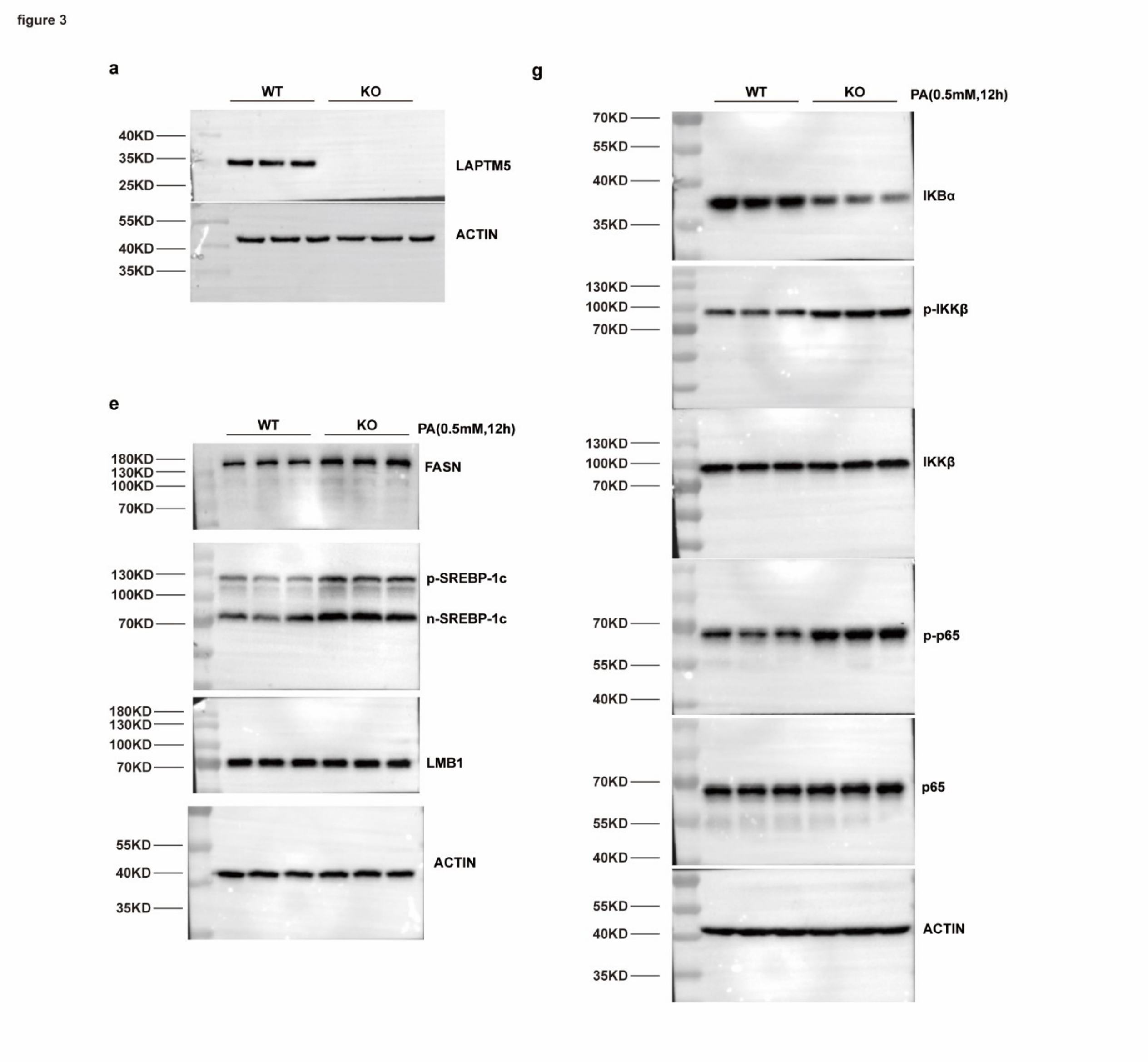

Uncropped gels for Western blot in figure 4

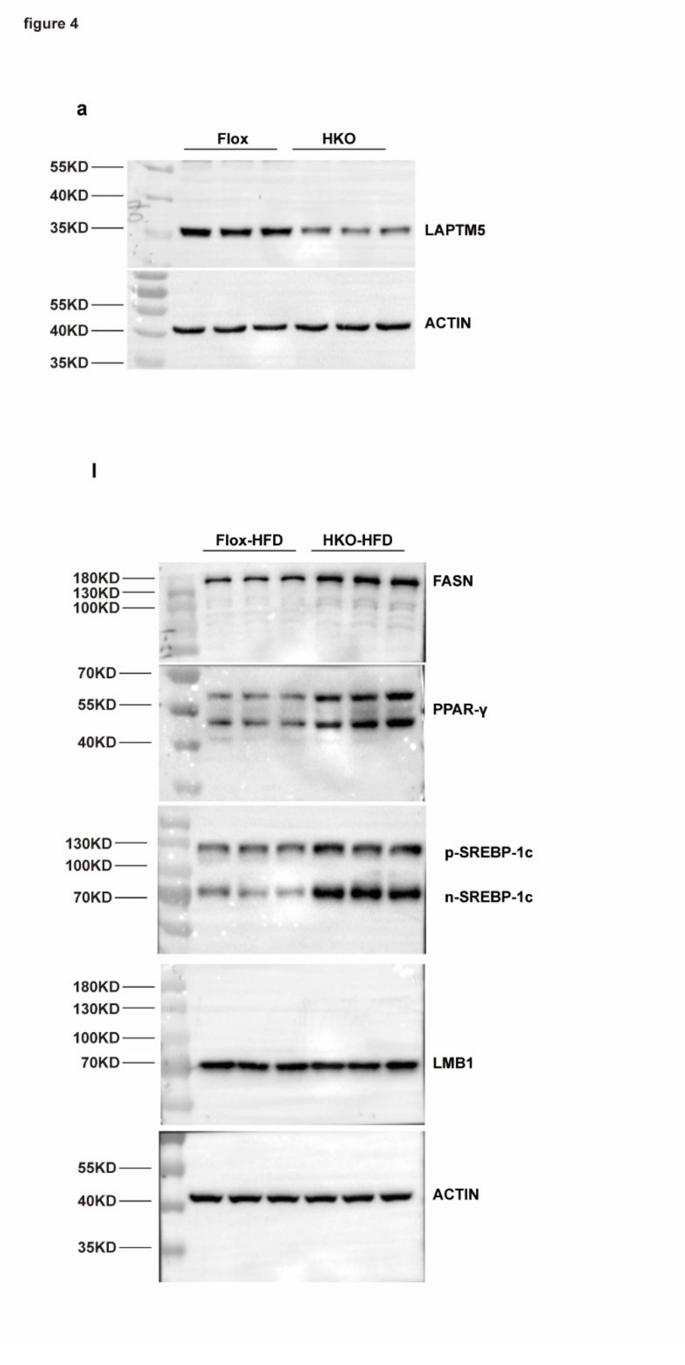

Uncropped gels for Western blot in figure 6

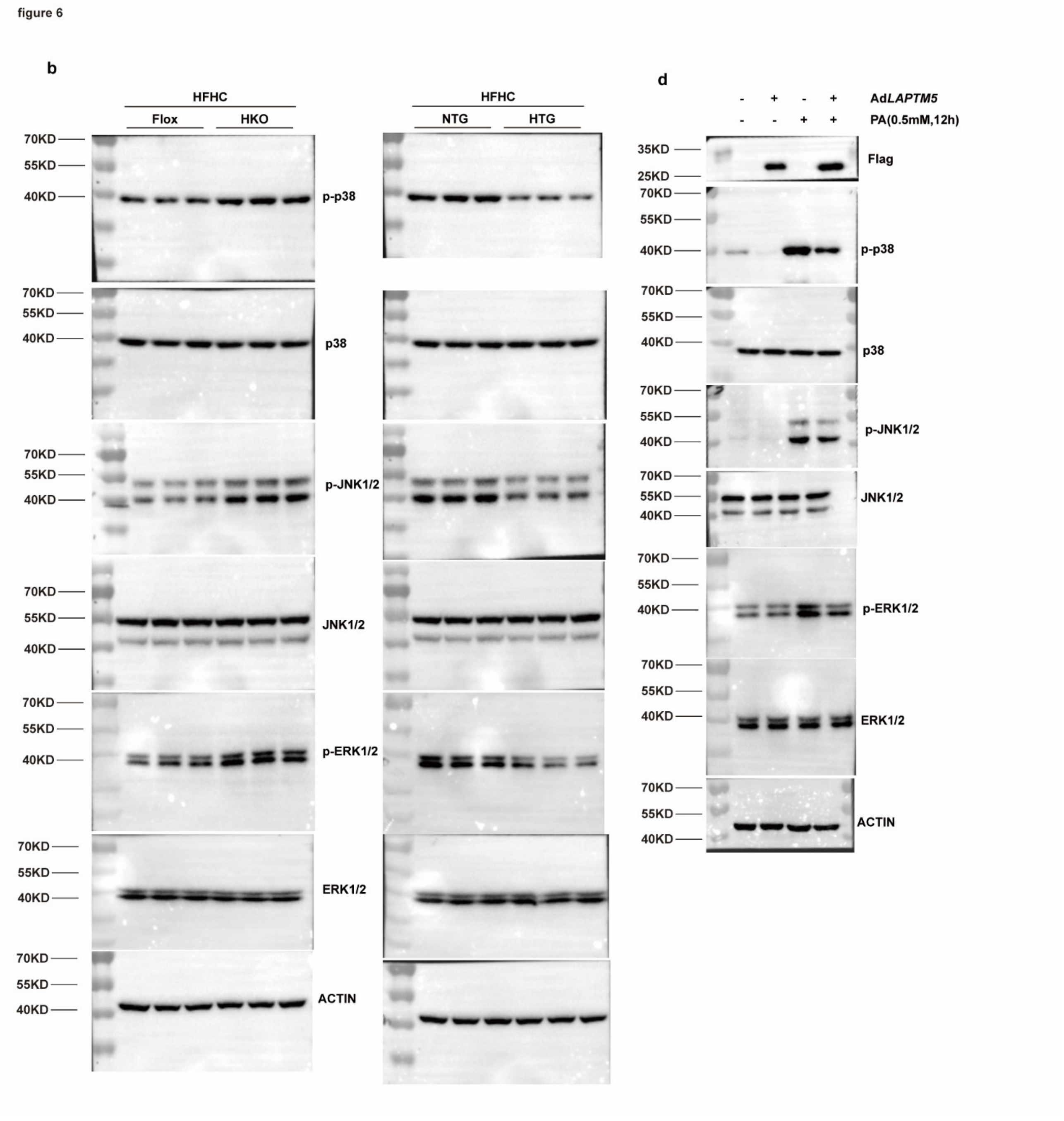

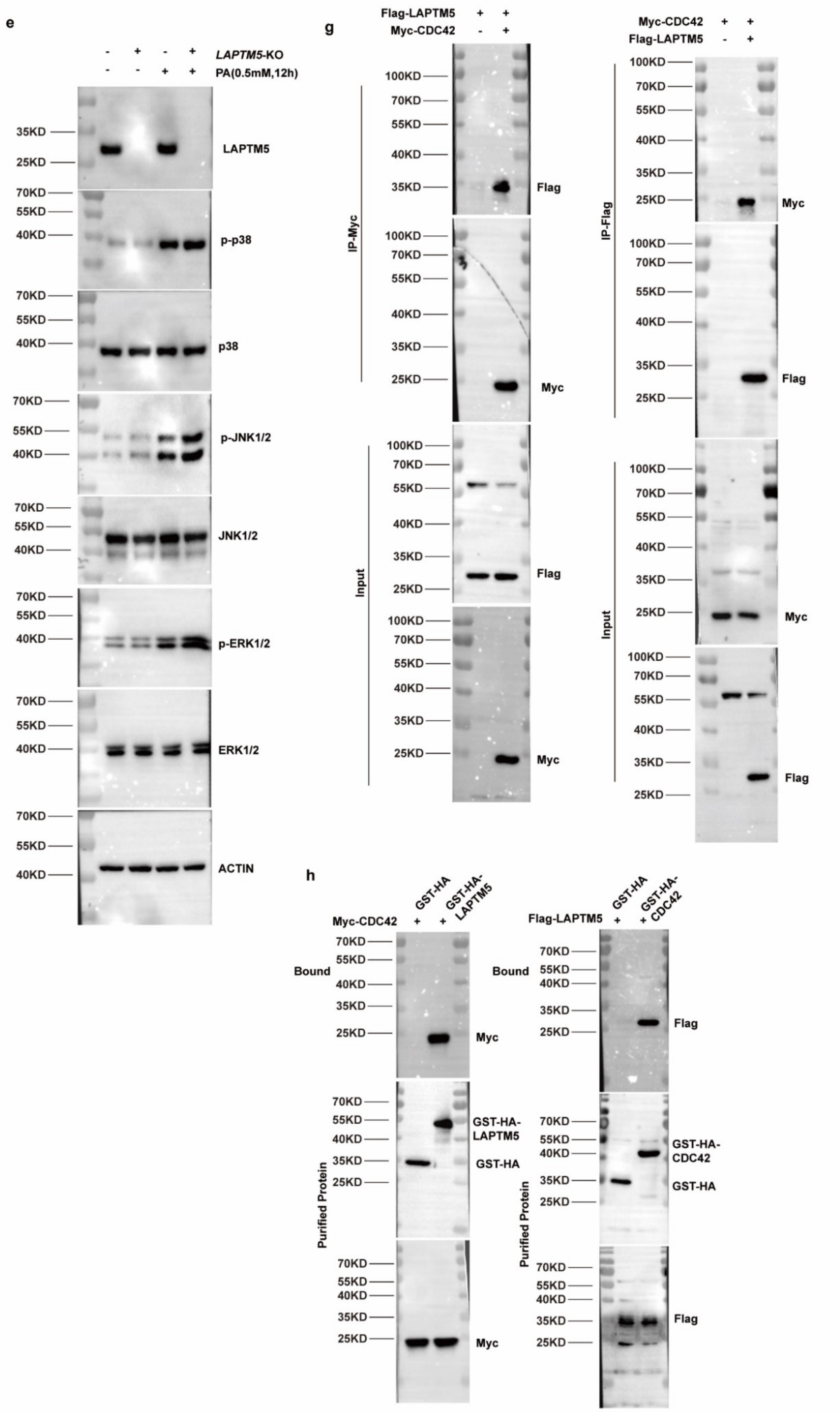
e
LAPTM5-KO
PA(0.5mM,12h)
35KD
25KD
LAPTM5
70KD
55KD
40KD
p-p38
p38
p-JNK1/2
JNK1/2
p-ERK1/2
ERK1/2
ACTIN
g
Flag-LAPTM5
Myc-CDC42
100KD
35KD
Flag
IP-Myc
Myc
Input
IP-Flag
h
GST-HA
GST-HA-LAPTM5
GST-HA-CDC42
Bound
Purified Protein
GST-HA-LAPTM5
GST-HA-CDC42
GST-HA

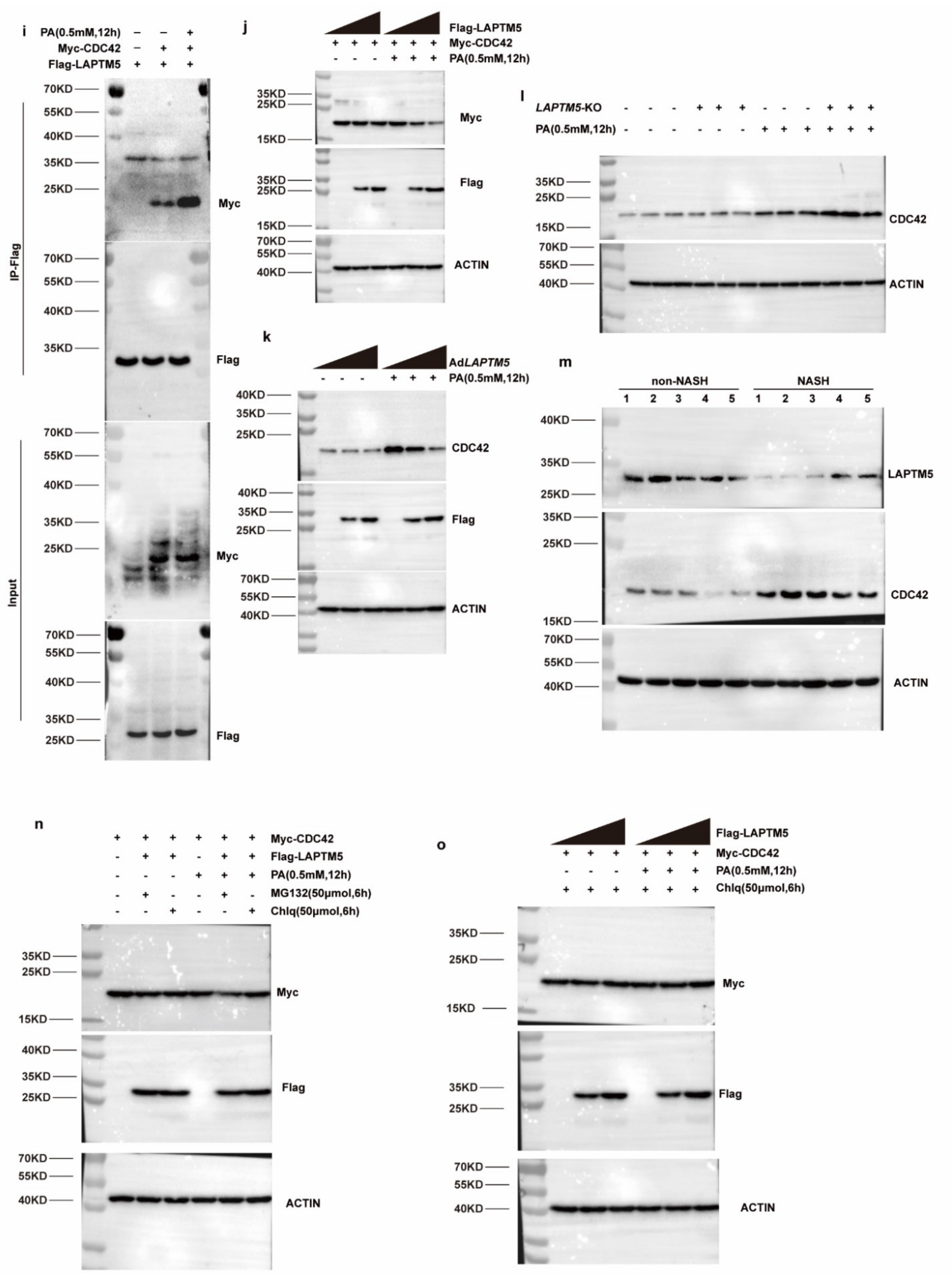
i
PA(0.5mM,12h)
Myc-CDC42
Flag-LAPTM5
IP-Flag
Input
Myc
Flag
j
Flag-LAPTM5
Myc-CDC42
PA(0.5mM,12h)
Myc
Flag
ACTIN
k
AdLAPTM5
PA(0.5mM,12h)
CDC42
Flag
ACTIN
l
LAPTM5-KO
PA(0.5mM,12h)
CDC42
ACTIN
m
non-NASH
NASH
LAPTM5
CDC42
ACTIN
n
Myc-CDC42
Flag-LAPTM5
PA(0.5mM,12h)
MG132(50μmol,6h)
Chlq(50μmol,6h)
Myc
Flag
ACTIN
o
Flag-LAPTM5
Myc-CDC42
PA(0.5mM,12h)
Chlq(50μmol,6h)
Myc
Flag
ACTIN

Uncropped gels for Western blot in figure 7

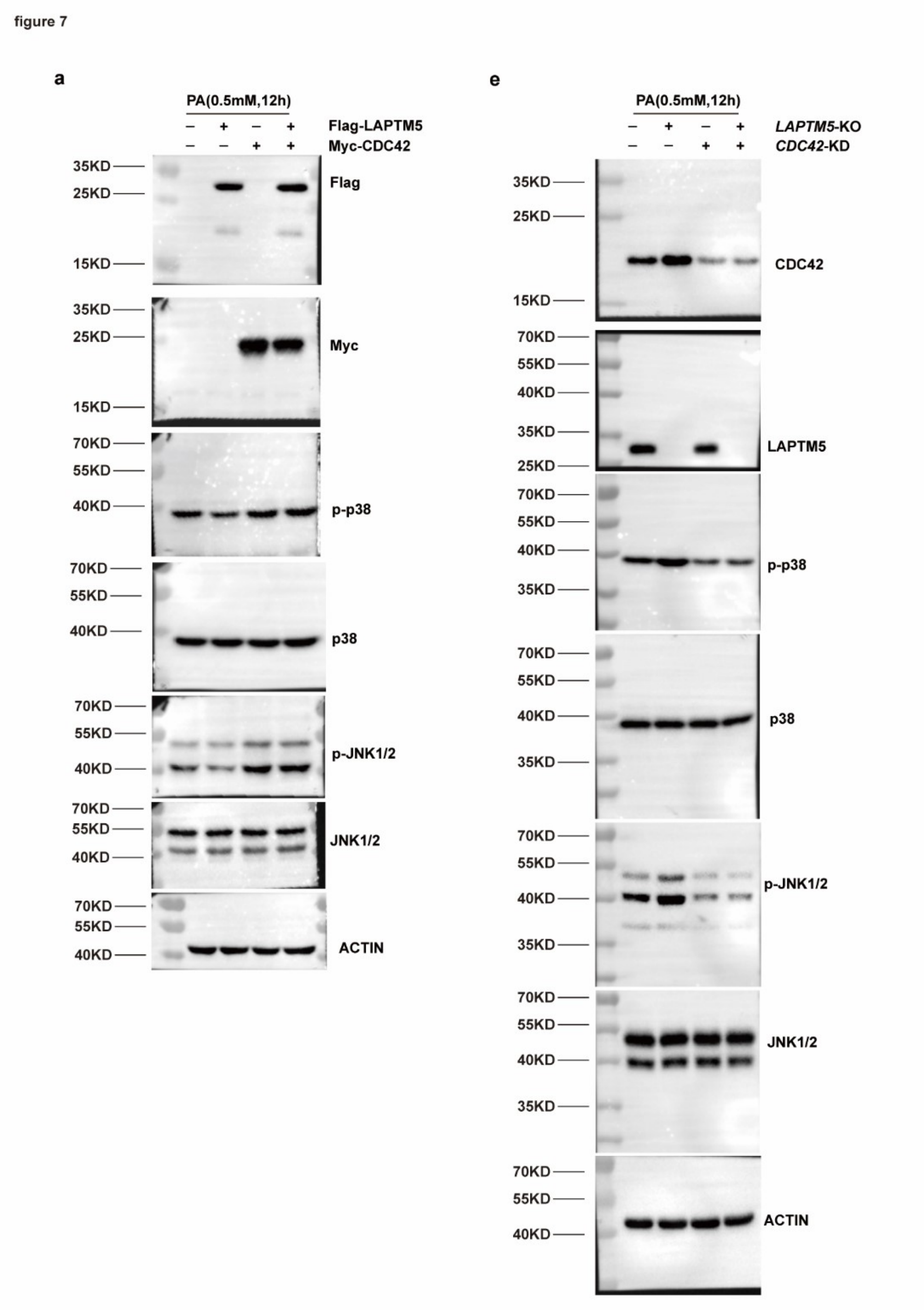

Uncropped gels for Western blot in figure 8

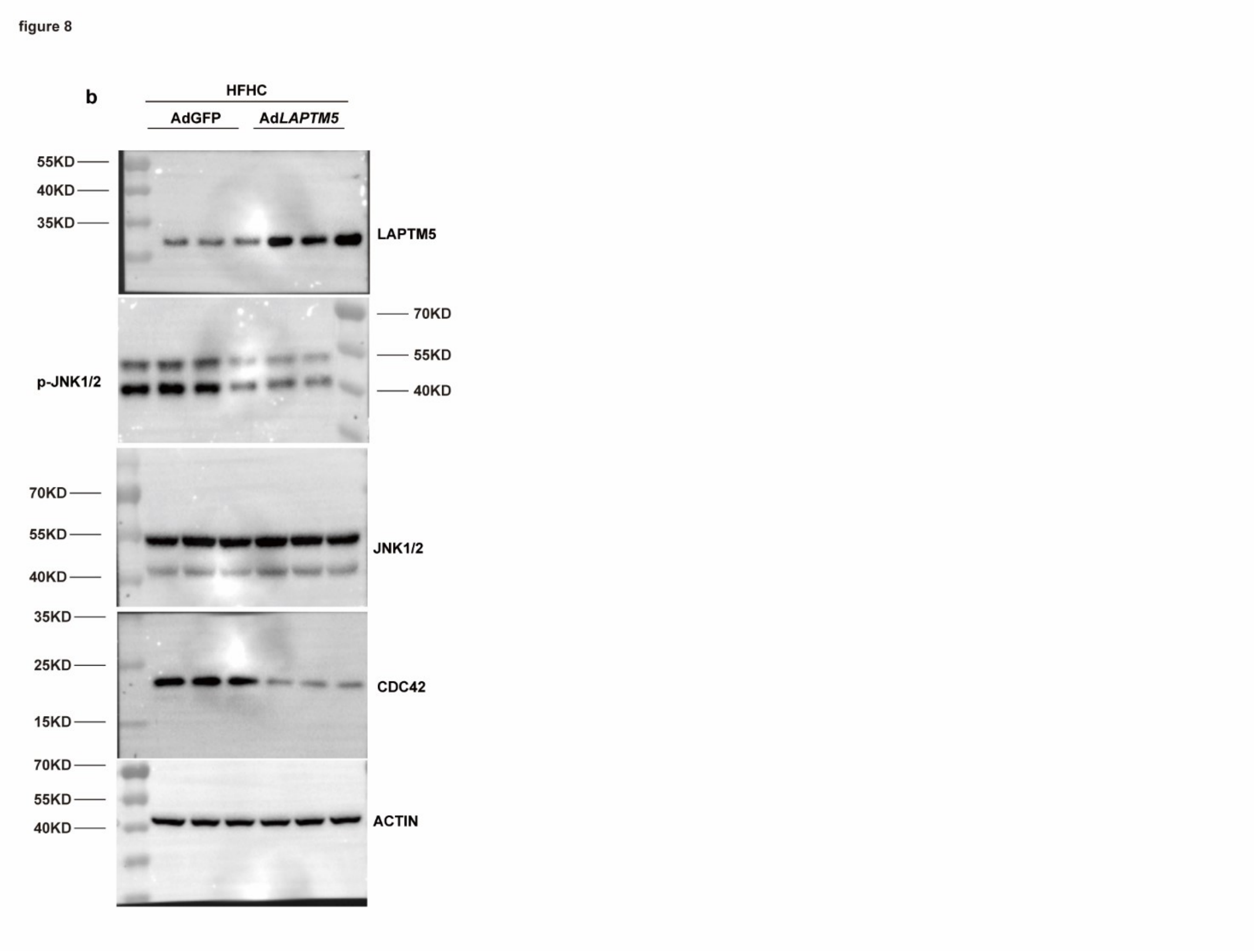

Uncropped gels for Western blot in supplementary figure 1

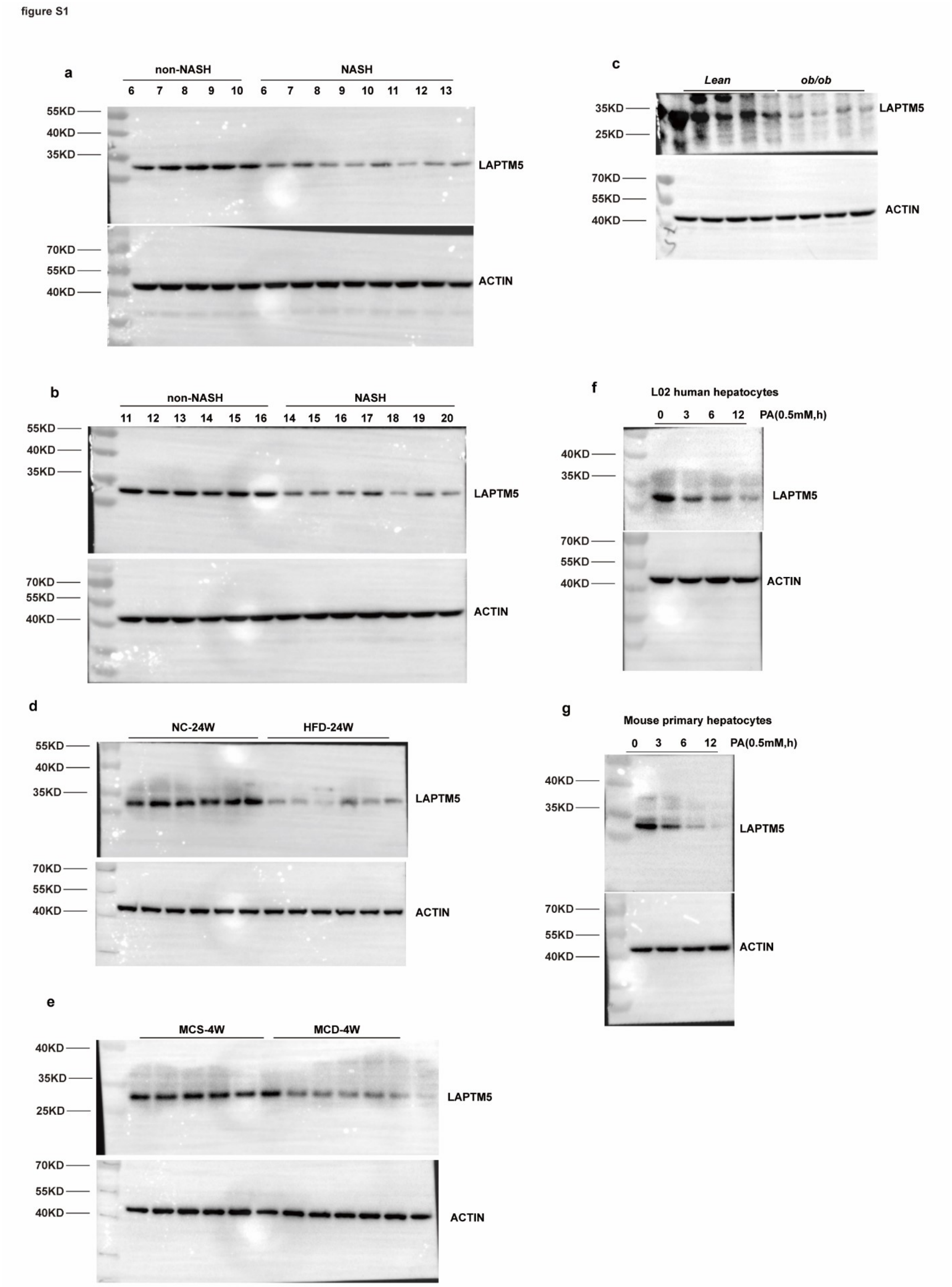

Uncropped gels for Western blot in supplementary figure 2-8

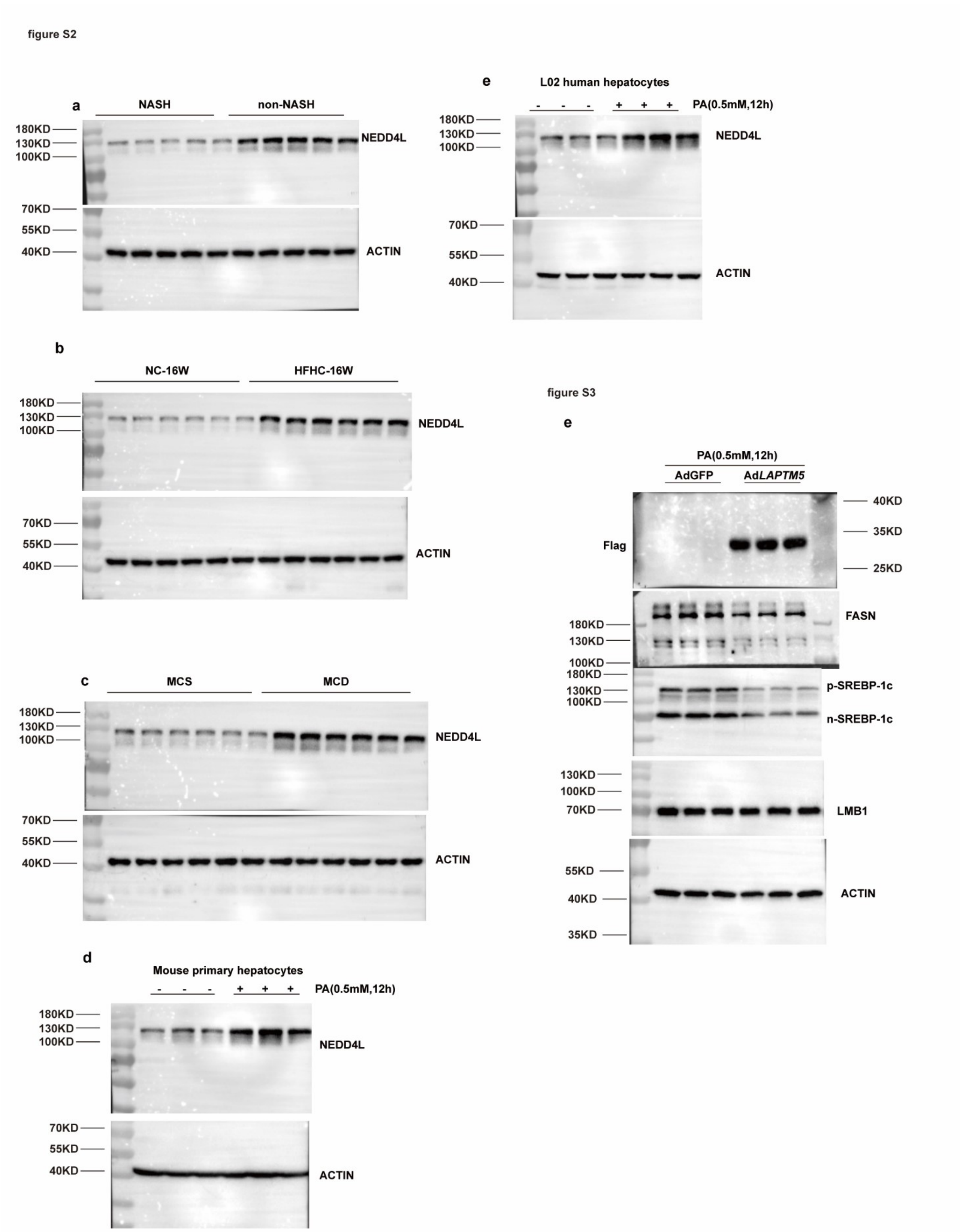

figure S3

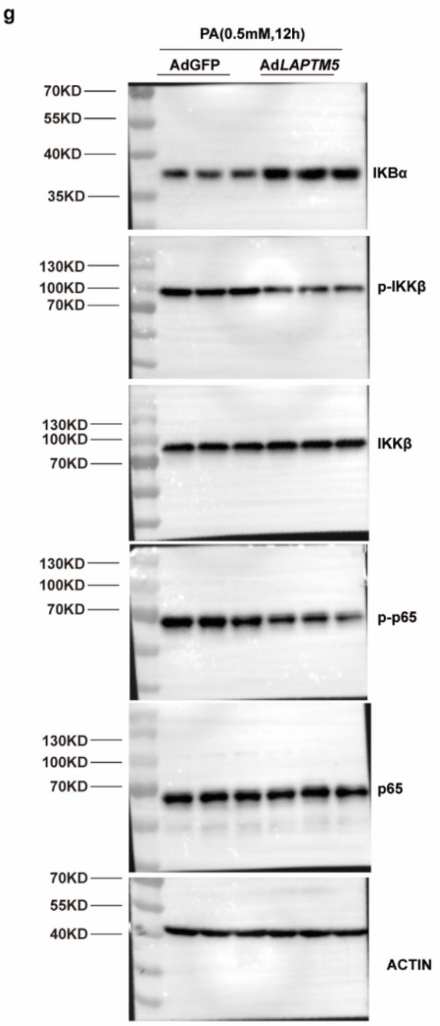

figure S8

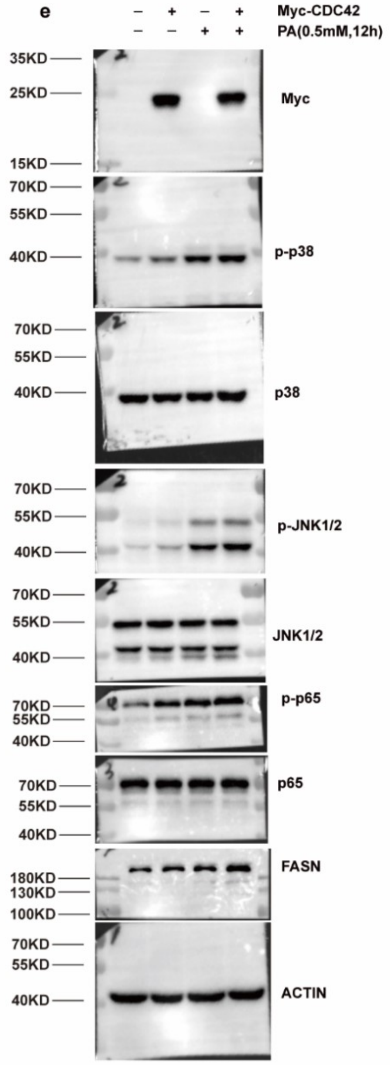

figure S7

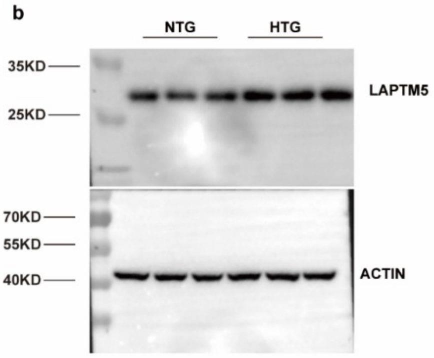

Supplementary Tables

Table S1. sgRNA target sequences for knockout mice construction

sgRNA	Target Sequence (5'-3')
sgRNA1	GGTTGCCTGGCCAAAGCATA_CGG
sgRNA2	CAAAGCTTGAATGGGTTGCA_GGG

Table S2. Primers for genotyping the liver specific knockout mice

Primer	Primer Sequence (5'-3')
P1	CACGTACCAATCCTGCTGCC
P2	CATCAGTAAGCGAGACCCAG
P3	ACCCAGAGTACCTACATTCC
P4	ACACCAGCCCAGAAATGC
P5	CACGGGGTGTTCAGGACAAC

Table S3. Primers for genotyping the liver specific transgenic mice

Primer	Primer Sequence (5'-3')
TG-check F1	GAGCGAGTCTTTCTGCACAC
TG-check R1	ATTTGAAGGGACAGGAAGGG

Table S4. Primers for overexpressing plasmid construction

Gene		Primer Sequence(5'-3')
Flag-LAPTM5	F	TCGGGTTTAAACGGATCCATGGACCCCCGCTTGTCCACTG
	R	GGGCCCTCTAGACTCGAGTCACACCTCTGAGTATGGGGGT
HA-NEDD4L	F	GTCCAGTGTGGTGGAATTATGGCGACCGGGCTCGGG
	R	TGGATATCTGCAGAATTTTAATCCACCCCTTCAAATCCTTG
HA-NEDD4	F	TCGGGTTTAAACGGATCCATGGCACAAAGCTTACGATTGC
	R	GGGCCCTCTAGACTCGAGCTAATCAACTCCATCAAAGCCCTG
HA-WWP2	F	TCGGGTTTAAACGGATCCATGGCATCTGCCAGCTCTAG
	R	GGGCCCTCTAGACTCGAGTTACTCCTGTCCAAAGCCCTC
HA-ITCH	F	AGATCTCTCGACGGATCCATGTCTGACAGTGGATCACAACTT
	R	TCCTTGTAATCCTCGAGTTACTCTTGTCCAAATCCTTCTGTTTC
HA-NEDD4L(C943S)	F	CAGAGCTCATACATCCTTTAATCGCC
	R	GGCGATTAAAGGATGTATGAGCTCTG
Myc-CDC42	F	TCGGGTTTAAACGGATCCATGCAGACAATTAAGTGTGTTGTTG
	R	GGGCCCTCTAGACTCGAGTCATAGCAGCACACACCTGC
GST-HA-LAPTM5	F	TCGGGTTTAAACGGATCCATGGACCCCCGCTTGTCCACTG
	R	GGGCCCTCTAGACTCGAGTCACACCTCTGAGTATGGGGGT
GST-HA-CDC42	F	TCGGGTTTAAACGGATCCATGCAGACAATTAAGTGTGTTGTTG
	R	GGGCCCTCTAGACTCGAGTCATAGCAGCACACACCTGC

Table S5. Primers for adenoviral plasmids construction

Gene		Primer Sequence(5'-3')
Ad-Flag-LAPTM5	F	TCCAGGTACCATTACTAGTATGGCCTCCCGTGCAGCG
	R	GTGTTTAAACCTGACTAGTTCACACTTCTGAGTATGGGGGTG
Ad-shCDC42	F1	CCGGCCGCTAAGTTATCCACAGACACTCGAGTGTCTGTGGATAACTTAGCGGTTTTTG
	R1	AATTCAAAAACCGCTAAGTTATCCACAGACACTCGAGTGTCTGTGGATAACTTAGCGG
	F2	CCGGCTGTCCAAAGACTCCTTTCTTCTCGAGAAGAAAGGAGTCTTTGGACAGTTTTTG
	R2	AATTCAAAAACTGTCCAAAGACTCCTTTCTTCTCGAGAAGAAAGGAGTCTTTGGACAG
	F3	CCGGTCTTGCTTGTTGGGACCCAAACTCGAGTTTGGGTCCCAACAAGCAAGATTTTTG
	R3	AATTCAAAAATCTTGCTTGTTGGGACCCAAACTCGAGTTTGGGTCCCAACAAGCAAGA

Table S6. Primers for qPCR

Gene		Primer Sequence(5'-3')
Laptm5	F	AGTGGCCTTTATCACCGTGC
	R	TGGCCGAATTCATGTGCTTC
Laptm5(Human)	F	CCTGAGCCTACTGATCGGC
	R	CAGGCACAGGAGATAGTCCA
Ppara	F	TATTCGGCTGAAGCTGGTGTAC
	R	CTGGCATTTGTTCCGGTTCT
Ppara (Human)	F	GTCATCACGGACACGCTTTCA
	R	CGGTCGCACTTGTCATACAC
Pparg	F	ATTCTGGCCCACCAACTTCGG
	R	TGGAAGCCTGATGCTTTATCCCCA
	F	CACTTCTGGAGACATCGCAAAC

Srebf1	R	ATGGTAGACAACAGCCGCATC
Srebf1 (Human)	F	ACAGTGACTTCCCTGGCCTAT
	R	GCATGGACGGGTACATCTTCAA
Scd1	F	TCTTCCTTATCATTGCCAACACCA
	R	GCGTTGAGCACCAGAGTGTATCG
Cd36	F	GACTGGGACCATTGGTGATGA
	R	AAGGCCATCTCTACCATGCC
Cd36 (Human)	F	TGCAAAGAAGGGAGACCTGTG
	R	GTTGACCTGCAGCCGTTTTG
Cpt1a	F	AGGACCCTGAGGCATCTATT
	R	ATGACCTCCTGGCATTCTCC
Cpt1a (Human)	F	TCCAGTTGGCTTATCGTGGTG
	R	CTAACGAGGGGTCGATCTTGG
Fasn	F	CTGCGGAAACTTCAGGAAATG
	R	GGTTCGGAATGCTATCCAGG
Il6	F	TAGTCCTTCCTACCCCAATTTCC
	R	TTGGTCCTTAGCCACTCCTTC
Il6 (Human)	F	GAGTAGTGAGGAACAAGCCAGA
	R	AAGCTGCGCAGAATGAGATGA
Il1b	F	CCGTGGACCTTCCAGGATGA
	R	GGGAACGTCACACACCAGCA
Mcp1	F	TACAAGAGGATCACCAGCAGC
	R	ACCTTAGGGCAGATGCAGTT
	F	CATCTTCTCAAAATTCGAGTGACAA

Tnf	R	TGGGAGTAGACAAGGTACAACCC
Tnf (Human)	F	TGGCGTGGAGCTGAGAGATA
	R	TGATGGCAGAGAGGAGGTTG
Cxcl2	F	GCGCCCAGACAGAAGTCATA
	R	CAGTTAGCCTTGCCTTTGTTCA
Cxcl10	F	ATGACGGGCCAGTGAGAATG
	R	ATGATCTCAACACGTGGGCA
Cxcl10 (Human)	F	GTGGCATTCAAGGAGTACCTC
	R	TGATGGCCTTCGATTCTGGATT
Il8	F	GGATCCTGATGCTCCATGGG
	R	CAGAAGCTTCATTGCCGGTG
Il8 (Human)	F	CTAGGACAAGAGCCAGGAAGAA
	R	GGGGTGGAAAGGTTTGGAGTA
Col1a1	F	TGCTAACGTGGTTCGTGACCGT
	R	ACATCTTGAGGTCGCGGCATGT
Col3a1	F	ACGTAAGCACTGGTGGACAG
	R	CCGGCTGGAAAGAAGTCTGA
Ctgf	F	TGACCCCTGCGACCCACA
	R	TACACCGACCCACCGAAGACACAG
a-SMA	F	CCCAGACATCAGGGAGTAATGG
	R	TCTATCGGATACTTCAGCGTCA
Hmgb1	F	GTTCTGAGTACCGCCCCAAA
	R	TAGGGCTGCTTGTCATCTGC
	F	CTCCTTCGGCCTTCTTCCTC

Hmgb1(Human)	R	GCTTGTCATCTGCAGCAGTG
Acox1	F	GTCTCCGTCATGAATCCCGA
	R	TGCGATGCCAAATTCCCTCA

Table S7. Antibodies

**Name**	**Citation**	**Supplier**	**Cat no.**	**Clone no.**
anti-b-Actin, dil:1/50000		Abclonal	Cat#AC026	
anti-LAPTM5,dil:1/200		Santa Cruz	Cat#sc134676	
anti-CD11b, dil: 1/100		Boster	Cat#BM3925	
anti-Ly6G, dil:1/100		abcam	Cat#AB42099	
anti-α-SMA, dil: 1/100		Cell Signaling Technology	Cat# 19245	
Goat anti-mouse IgG (H+L), dil:1/5000		Jackson	Cat#115-035-003	
Goat anti-rabbit IgG (H+L), dil:1/5000		Jackson	Cat#111-035-003	
anti-phospho-JNK1/2(Thr183/Tyr185), dil:1/1000		Cell Signaling Technology	Cat#4668	
anti-JNK1/2, dil:1/1000		Cell Signaling Technology	Cat#9258	

anti-phospho-p38 (Thr180/Tyr182), dil:1/1000		Cell Signaling Technology	Cat#4511	
anti-p38, dil:1/1000		Cell Signaling Technology	Cat#9212	
anti-HA, dil:1/1000		Cell Signaling Technology	Cat#3724	
anti-Flag, dil:1/1000		Proteintech	Cat#20543-1-AP	
anti-Myc,dil:1/1000		Cell Signaling Technology	Cat# 2278	
anti-HA, dil:1/500		MBL	Cat#M180-3	
anti-Flag, dil:1/500		MBL	Cat#M185	
anti-Myc, dil:1/500		MBL	Cat#M047-3	
anti-SREBP-1c, dil: 1/1000		Abcam	Cat#ab28481	
anti-Fasn,dil:1/1000		Abcam	Cat# ab15285	
anti-PPAR-γ,dil:1/1000		Cell Signaling Technology	Cat# 2435	
anti-phospho-p65 (Ser536), dil:1/1000		Cell Signaling Technology	clone 93H1, Cat#3033	
anti-p65, dil:1/1000		Cell Signaling Technology	clone C22B4, Cat#4764	
anti-Rac1,dil:1/1000		Abclonal	Cat# A5080	

anti-Cdc42, dil:1/1000		Cell Signaling Technology	Cat# 2466	
anti-LAMP1, dil:1/200		Developmental Studies Hybridoma Bank	Cat# 1d4b	
anti-Cdc42, dil:1/200		Abclonal	Cat# A1188	
Alexa Flour 568 goat anti-rabbit IgG (H+L), dil:1/200		Invitrogen	Cat#A11036	
Alexa Flour 568 goat anti-rabbit IgG (H+L), dil:1/200		Invitrogen	Cat#A11029	

Table S8. Cell lines

**Name**	**Citation**	**Supplier**	**Cat no.**	**Passage no.**	**Authentication test method**
Human: L02 (HL-7702)		Type Culture Collection of the Chinese Academy of Sciences	Cat#GNHu 6		

Human: HEK293T		Type Culture Collection of the Chinese Academy of Sciences	Cat#GNHu17		
Human: HEK293		Type Culture Collection of the Chinese Academy of Sciences	Cat#GNHu43		

Table S9. Software

**Software name**	**Manufacturer**	**Version**
DESeq2 1.2.10	Love et al., 2014	
SPSS statistics 19.0	IBM Corp	
GraphPad Prism 7	Graphpad Software	
FIJI running ImageJ, version 1.52p	ImageJ	
GSEA 3.0	Subramanian et al., 2005	

Table S10. Other (e.g. drugs, proteins, vectors etc.)

Oil Red O	Sigma-Aldrich	Cat#O0625
Nile Red	Fanbo Biochemicals	Cat#22190
Chloroquine (NSC-187208)	Selleck Chemicals	Cat#S6999
MG-132	Selleck Chemicals	Cat#S2619
Fetal Bovine Serum (FBS)	Bio-One Biotechnology	Cat#F05-001-B160216
Penicillin-Streptomycin	GIBCO	Cat#15140-122
Palmitic acid (PA)	Sigma-Aldrich	Cat#P0500
Oleic acid (OA)	Sigma-Aldrich	Cat#O-1008
Pentobarbital sodium	Sigma-Aldrich	Cat#P3761
DMEM	GIBCO	Cat#C11995500BT
Liver Perfusion Medium	Thermo Fisher Scientific	Cat#17701-038
Liver Digestion Medium	Thermo Fisher Scientific	Cat#17701-034
Passive Lysis 5X Buffer	Promega	Cat#E1941
Trizol	Sigma-Aldrich	Cat#T9424
Insulin	Thermo Fisher Scientific	Cat#12585-014
Formalin	Thermo Fisher Scientific	Cat#23-305-510
RIPA buffer	Beyotime Biotechnology	Cat#P0013E
Triton X-100	Sigma-Aldrich	Cat#T8787
Trypsin	GIBCO	Cat#27250-018
DAPI	Invitrogen	Cat#S36939
PMSF	Beyotime Biotechnology	Cat#ST506

SYBR Green PCR Master Mix	Roche	Cat#04887352001
Picrosirius Red	Hede Biotechnology	Cat#26357-02
cOmplete Protease Inhibitor Cocktail	Roche	Cat#04693132001
PhosStop phosphatase inhibitor	Roche	Cat#4906837001
Hematoxylin	Google biology	Cat#G1004
Eosin	BASO	Cat#BA-4024
Tissue-Tek O.C.T. Compound	Servicebio	Cat#4583
Collagenase IV	GIBCO	Cat#17104019
DNA Purification Kit	Beyotime Biotechnology	Cat#D0033
BCA protein assay kit	Thermo Fisher Scientific	Cat#23225
LabAsssay Cholesterol	Wako	Cat#294-65801
LabAsssay Triglyceride	Wako	Cat#290-63701
Transcriptor first-strand cDNA synthesis kit	Roche	Cat#04896866001
Mouse high fat diet	Huafukang Bioscience	Cat#H10060
Mouse high fat high cholesterol diet	TrophicDiet	Cat#TP26304
Mouse methionine and choline deficient diet	TrophicDiet	Cat#TP3005G

Mouse methionine and choline sufficient diet	TrophicDiet	Cat#TP3005GS
Mouse normal chow diet	Xietong Shengwu	Cat#1010001
PVDF membranes	Millipore	Cat#IPVH00010
GloMax 20/20 Luminometer	Promega	Cat#E5311
