# Supplementary material for: Lysosomal-associated protein transmembrane 5 ameliorates non-alcoholic steatohepatitis by promoting the degradation of CDC42 in mice
Source: Nat Commun. 2023 May 8;14:2654. doi: 10.1038/s41467-023-37908-9 (PMC10167344; doi:10.1038/s41467-023-37908-9)
Supplement: Supplementary file 3 — Reporting Summary [file 41467_2023_37908_MOESM3_ESM.pdf]

nature portfolio

Corresponding author(s): Jiahong Xia

Last updated by author(s): Mar 21, 2023

Reporting Summary

Nature Portfolio wishes to improve the reproducibility of the work that we publish. This form provides structure for consistency and transparency in reporting. For further information on Nature Portfolio policies, see our Editorial Policies and the Editorial Policy Checklist.

Statistics

For all statistical analyses, confirm that the following items are present in the figure legend, table legend, main text, or Methods section.

n/a	Confirmed	
☐	☒	The exact sample size (*n*) for each experimental group/condition, given as a discrete number and unit of measurement
☐	☒	A statement on whether measurements were taken from distinct samples or whether the same sample was measured repeatedly
☐	☒	The statistical test(s) used AND whether they are one- or two-sided<br>*Only common tests should be described solely by name; describe more complex techniques in the Methods section.*
☒	☐	A description of all covariates tested
☐	☒	A description of any assumptions or corrections, such as tests of normality and adjustment for multiple comparisons
☐	☒	A full description of the statistical parameters including central tendency (e.g. means) or other basic estimates (e.g. regression coefficient) AND variation (e.g. standard deviation) or associated estimates of uncertainty (e.g. confidence intervals)
☐	☒	For null hypothesis testing, the test statistic (e.g. *F*, *t*, *r*) with confidence intervals, effect sizes, degrees of freedom and *P* value noted<br>*Give P values as exact values whenever suitable.*
☒	☐	For Bayesian analysis, information on the choice of priors and Markov chain Monte Carlo settings
☒	☐	For hierarchical and complex designs, identification of the appropriate level for tests and full reporting of outcomes
☒	☐	Estimates of effect sizes (e.g. Cohen's *d*, Pearson's *r*), indicating how they were calculated

Our web collection on statistics for biologists contains articles on many of the points above.

Software and code

Policy information about availability of computer code

Data collection	Image Lab 6.1 software, ImageJ 1.8.0 software.
Data analysis	SPSS 21.0 software, Java GSEA 3.0 software, DESeq2 1.2.10 software, StringTie 1.3.3b software, SAMtools 1.4 software, HISAT2 2.1.0 software.

For manuscripts utilizing custom algorithms or software that are central to the research but not yet described in published literature, software must be made available to editors and reviewers. We strongly encourage code deposition in a community repository (e.g. GitHub). See the Nature Portfolio guidelines for submitting code & software for further information.

Data

Policy information about availability of data

All manuscripts must include a data availability statement. This statement should provide the following information, where applicable:

- Accession codes, unique identifiers, or web links for publicly available datasets
- A description of any restrictions on data availability
- For clinical datasets or third party data, please ensure that the statement adheres to our policy

Provide your data availability statement here.

Human research participants

Policy information about studies involving human research participants and Sex and Gender in Research.

Reporting on sex and gender	Sex and gender were not considered in our study design.
Population characteristics	The liver samples from patients are obtained from non-NASH group for 16 people, NASH group for 20 people. For the non-NASH group, there are 9 female patients and 7 male patients with age ranges from 27 to 67 years old. For the NASH group, there are 13 female patients and 7 male patients with age ranges from 19 to 40 years old.
Recruitment	We obtained human steatotic liver samples from the patients with simple steatosis or NASH who had undergone liver biopsy or liver transplantation. The non-steatotic liver samples were obtained from the healthy regions of livers from donors who had undergone liver resection because of hepatic cyst or liver hemangioma. Individuals enrolled in this study were excluded from hepatitis virus infection, drug abuse or excessive alcohol consumption (> 140 g for men or > 70 g for women, per week).Two pathologists independently used the NASH-CRN scoring system to evaluate the NAFLD activity score (NAS) in a blinded fashion. The non-NASH samples possessed an NAS of 0. The NASH samples possessed a NAS > 4, or NAS at 3-4 but exhibiting fibrosis. However, unavoidable operational errors at the time of sampling may cause results to be inconsistent with the actual degree of disease in patients, thus affecting the results of the study and slightly biased from the actual situation.
Ethics oversight	Ethics committee of Renmin Hospital of Wuhan University

Note that full information on the approval of the study protocol must also be provided in the manuscript.

Field-specific reporting

Please select the one below that is the best fit for your research. If you are not sure, read the appropriate sections before making your selection.

☒ Life sciences ☐ Behavioural & social sciences ☐ Ecological, evolutionary & environmental sciences

For a reference copy of the document with all sections, see nature.com/documents/nr-reporting-summary-flat.pdf

Life sciences study design

All studies must disclose on these points even when the disclosure is negative.

Sample size	The sample size in our study are determined by referring to literature on NASH research previously published in Nature Communications. Doi.org/10.1038/s41467-021-27539-3; Doi.org/10.1038/s41467-020-14384-z.
Data exclusions	No data were excluded from the analysis.
Replication	The experiments in our study have all been performed independently at least 3 times, and make sure the experimental results are consistent.
Randomization	For mice in our research, littermate-born C57BL/6 background mice were randomly assigned and given a different diet to simulate as mentioned in the article to mimic NASH. Except for experiments involving mice, the samples were randomly divided into experimental and control groups.
Blinding	The investigators were blinded to group allocation during data collection and analysis.

Reporting for specific materials, systems and methods

We require information from authors about some types of materials, experimental systems and methods used in many studies. Here, indicate whether each material, system or method listed is relevant to your study. If you are not sure if a list item applies to your research, read the appropriate section before selecting a response.

Materials & experimental systems

n/a	Involved in the study	
☐	☒	Antibodies
☐	☒	Eukaryotic cell lines
☒	☐	Palaeontology and archaeology
☐	☒	Animals and other organisms
☒	☐	Clinical data
☒	☐	Dual use research of concern

Methods

n/a	Involved in the study	
☒	☐	ChIP-seq
☒	☐	Flow cytometry
☒	☐	MRI-based neuroimaging

Antibodies

Antibodies used	Anti-b-Actin Rabbit mAb(dil:1/50000, Abclonal, Cat#AC026). All the information about the antibodies used in our study are collated and listed in the Reagents and antibodies section of the Supplementary files which has been uploaded.
Validation	The antibody is well validated and the target band is clear and free of heterobands. The validation statements on the manufacturer's website, relevant citations, and antibody profile in online databases can be checked by visiting the company's official website. All the information about antibodies and experimental data and uncropped versions of any gels or blots have been collated and uploaded as Source Data and listed in the Supplementary files which has been uploaded.

Eukaryotic cell lines

Policy information about cell lines and Sex and Gender in Research

Cell line source(s)	Human hepatocyte L02, human embryonic kidney 293, and 293T cell lines were purchased from the Type Culture Collection of the Chinese Academy of Sciences (Shanghai, China).
Authentication	We perform cell line identification by STR genotyping.
Mycoplasma contamination	All the cell lines were examined for mycoplasma contamination, and the results were negative. All the cell lines in our laboratory were passaged no more than 30 times after resuscitation and routinely tested for mycoplasma contamination by PCR.
Commonly misidentified lines (See ICLAC register)	There is no commonly misidentified cell lines in the study.

Animals and other research organisms

Policy information about studies involving animals; ARRIVE guidelines recommended for reporting animal research, and Sex and Gender in Research

Laboratory animals	Adult 8 to 10 week-old C57BL/6 male mice and weighing 22–30g, were housed under pathogen-free conditions in a temperature-controlled environment at 22–24°C, humidity-controlled environment at 40%-70% under a 12-hour light/dark cycle with ad libitum access to water and food.
Wild animals	The study did not involve wild animals.
Reporting on sex	Only male mice participated in this experimental study.
Field-collected samples	The study did not involve samples collected from the field.
Ethics oversight	The Institutional Animal Care and Use Committees of Tongji Medical College and the Huazhong University of Science and Technology approved the animal protocols used in this study.

Note that full information on the approval of the study protocol must also be provided in the manuscript.
