# Supplementary material for: Lysosomal-associated protein transmembrane 5 ameliorates non-alcoholic steatohepatitis by promoting the degradation of CDC42 in mice
Source: Nat Commun. 2023 May 8;14:2654. doi: 10.1038/s41467-023-37908-9 (PMC10167344; doi:10.1038/s41467-023-37908-9)

figure 1

g

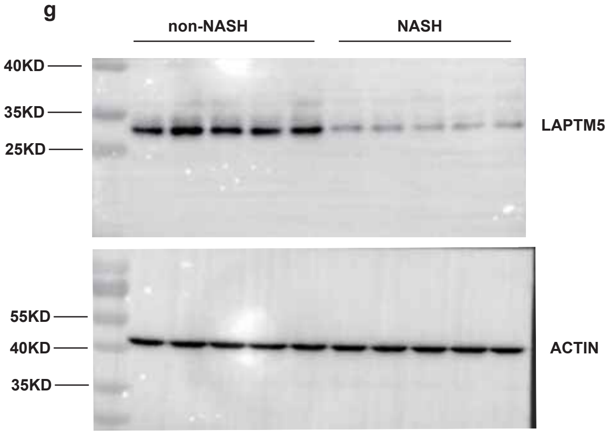
non-NASH
NASH
40KD
35KD
25KD
LAPTM5
55KD
40KD
35KD
ACTIN

j

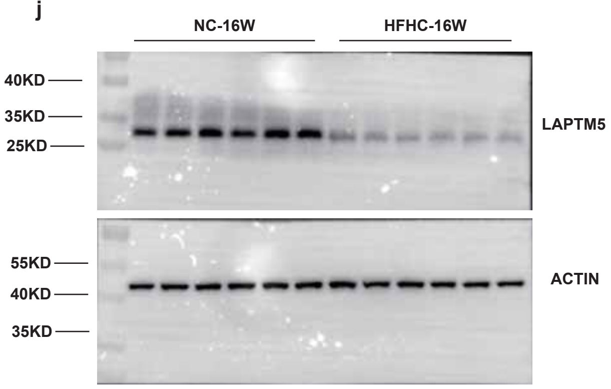
NC-16W
HFHC-16W
40KD
35KD
25KD
LAPTM5
55KD
40KD
35KD
ACTIN

figure 2

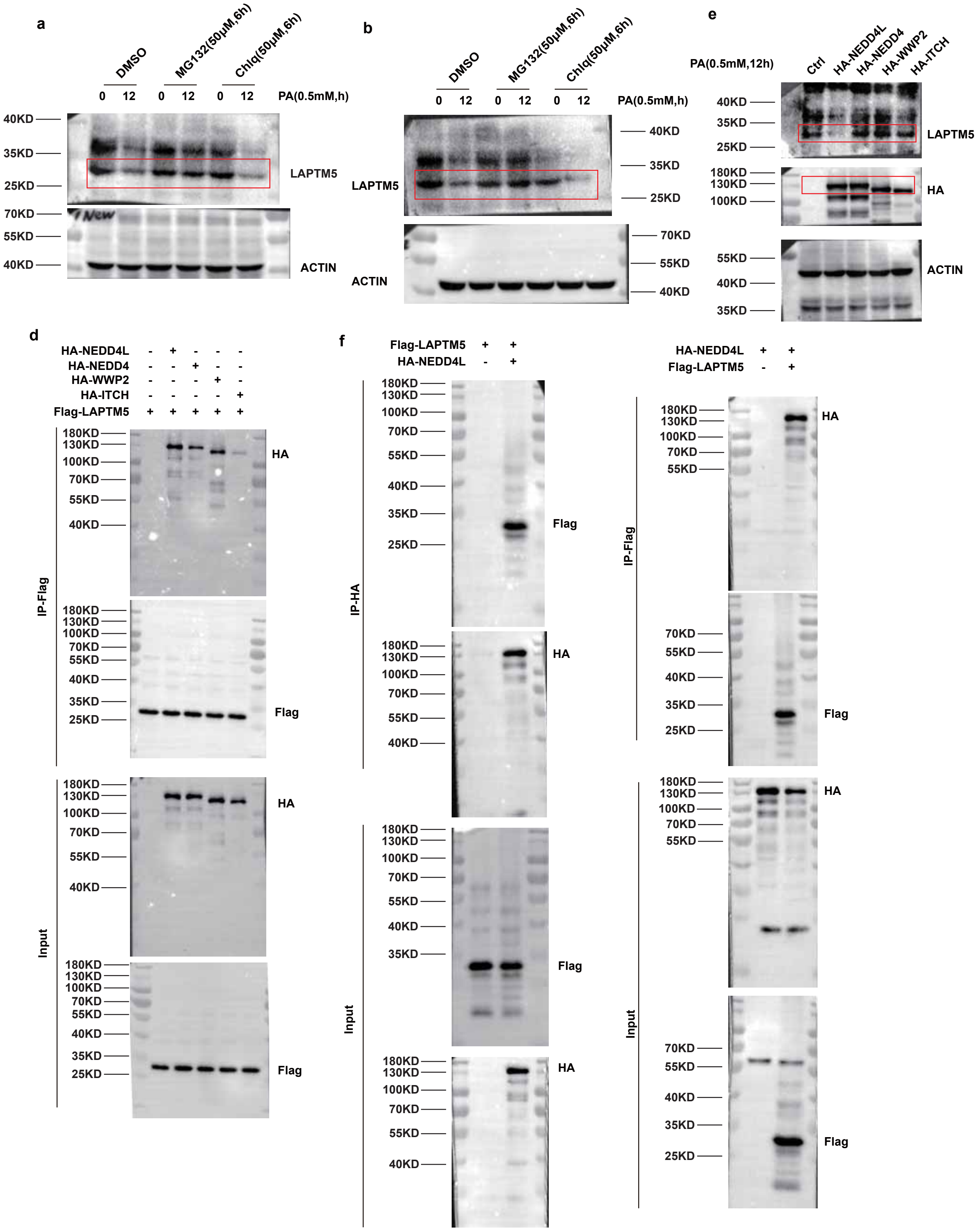

a
DMSO
MG132(50μM,6h)
Chlq(50μM,6h)
0 12 0 12 0 12 PA(0.5mM,h)
40KD
35KD
25KD
LAPTM5
70KD
55KD
40KD
ACTIN
b
LAPTM5
ACTIN
e
PA(0.5mM,12h)
Ctrl
HA-NEDD4L
HA-NEDD4
HA-WWP2
HA-ITCH
HA
180KD
130KD
100KD
d
HA-NEDD4L
HA-NEDD4
HA-WWP2
HA-ITCH
Flag-LAPTM5
IP-Flag
Input
Flag
f
Flag-LAPTM5
HA-NEDD4L
IP-HA

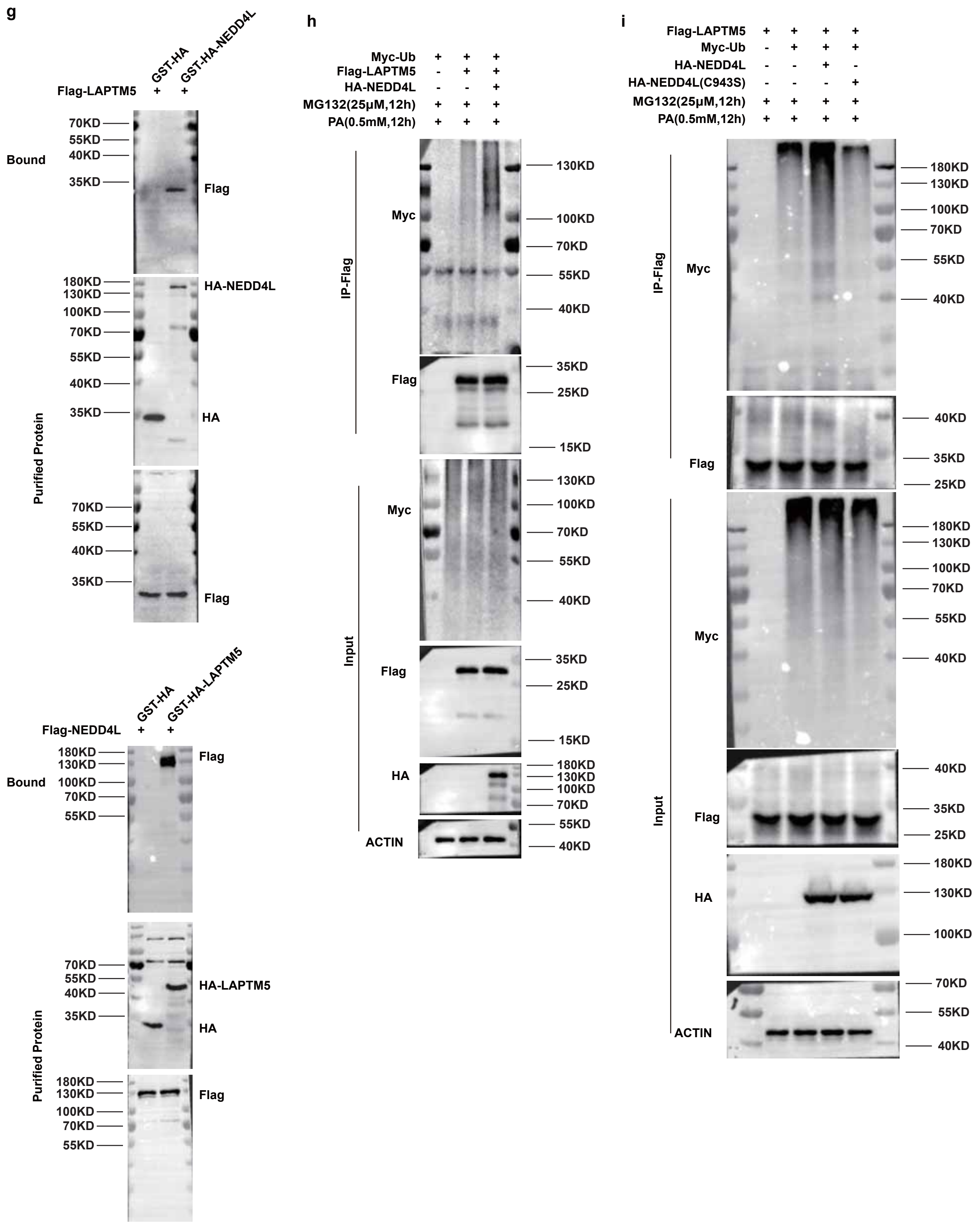
g
GST-HA
GST-HA-NEDD4L
Flag-LAPTM5 + +
Bound
70KD
55KD
40KD
35KD
Flag
180KD
130KD
100KD
70KD
55KD
40KD
35KD
HA-NEDD4L
HA
Purified Protein
Flag
GST-HA
GST-HA-LAPTM5
Flag-NEDD4L + +
Bound
Flag
HA-LAPTM5
HA
Purified Protein
h
Myc-Ub + + +
Flag-LAPTM5 - + +
HA-NEDD4L - - +
MG132(25μM,12h) + + +
PA(0.5mM,12h) + + +
IP-Flag
Myc
Flag
Input
HA
ACTIN
130KD
100KD
70KD
55KD
40KD
35KD
25KD
15KD
180KD
i
Flag-LAPTM5 + + + +
Myc-Ub - + + +
HA-NEDD4L - - + -
HA-NEDD4L(C943S) - - - +
MG132(25μM,12h) + + + +
PA(0.5mM,12h) + + + +
IP-Flag
Myc
Flag
Input
HA
ACTIN

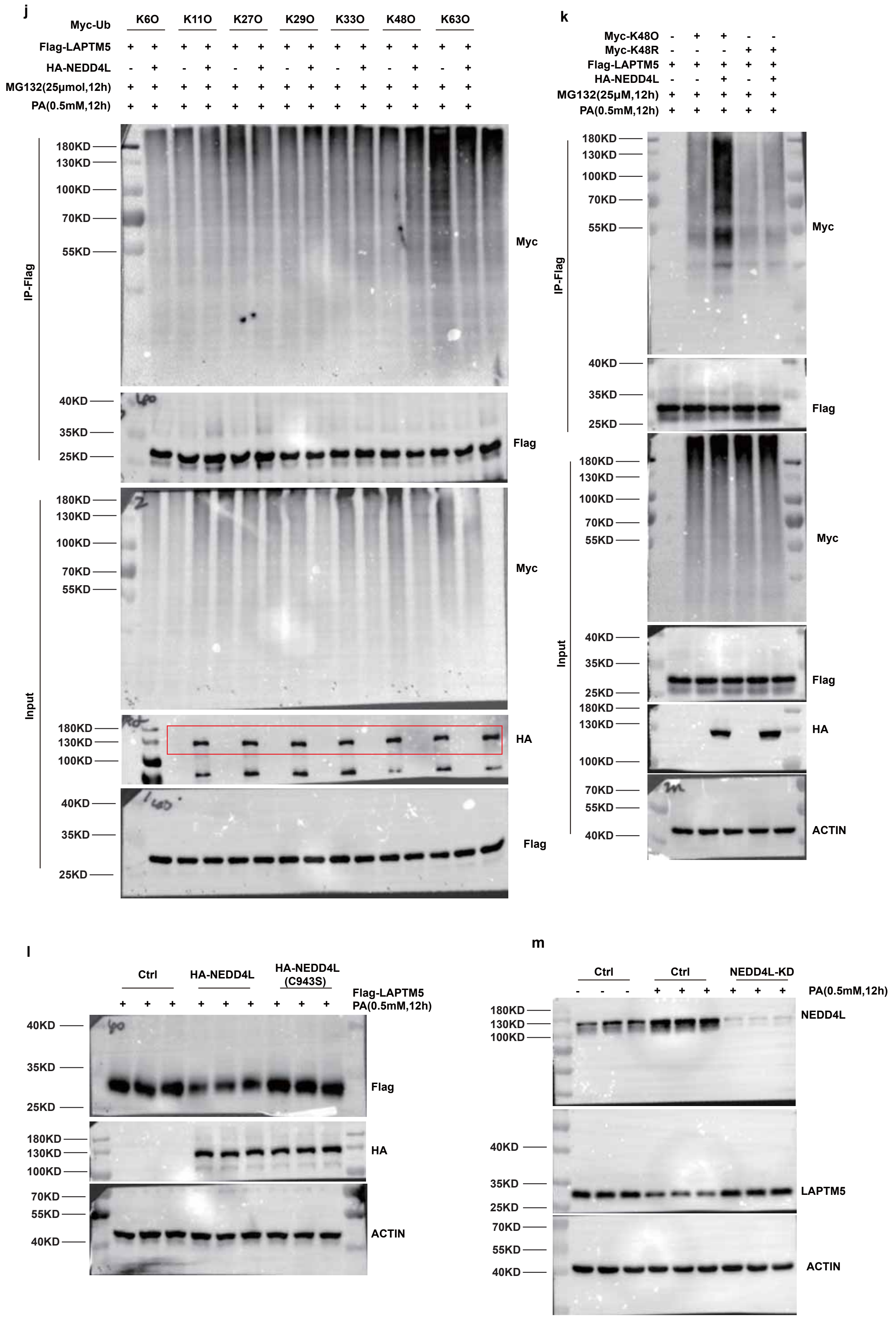

j
Myc-Ub
K6O
K11O
K27O
K29O
K33O
K48O
K63O
Flag-LAPTM5
HA-NEDD4L
MG132(25μmol,12h)
PA(0.5mM,12h)
IP-Flag
Input
180KD
130KD
100KD
70KD
55KD
40KD
35KD
25KD
Myc
Flag
HA
k
Myc-K48O
Myc-K48R
Flag-LAPTM5
HA-NEDD4L
MG132(25μM,12h)
PA(0.5mM,12h)
ACTIN
l
Ctrl
HA-NEDD4L
HA-NEDD4L (C943S)
Flag-LAPTM5
PA(0.5mM,12h)
m
Ctrl
NEDD4L-KD
NEDD4L
LAPTM5

figure 3

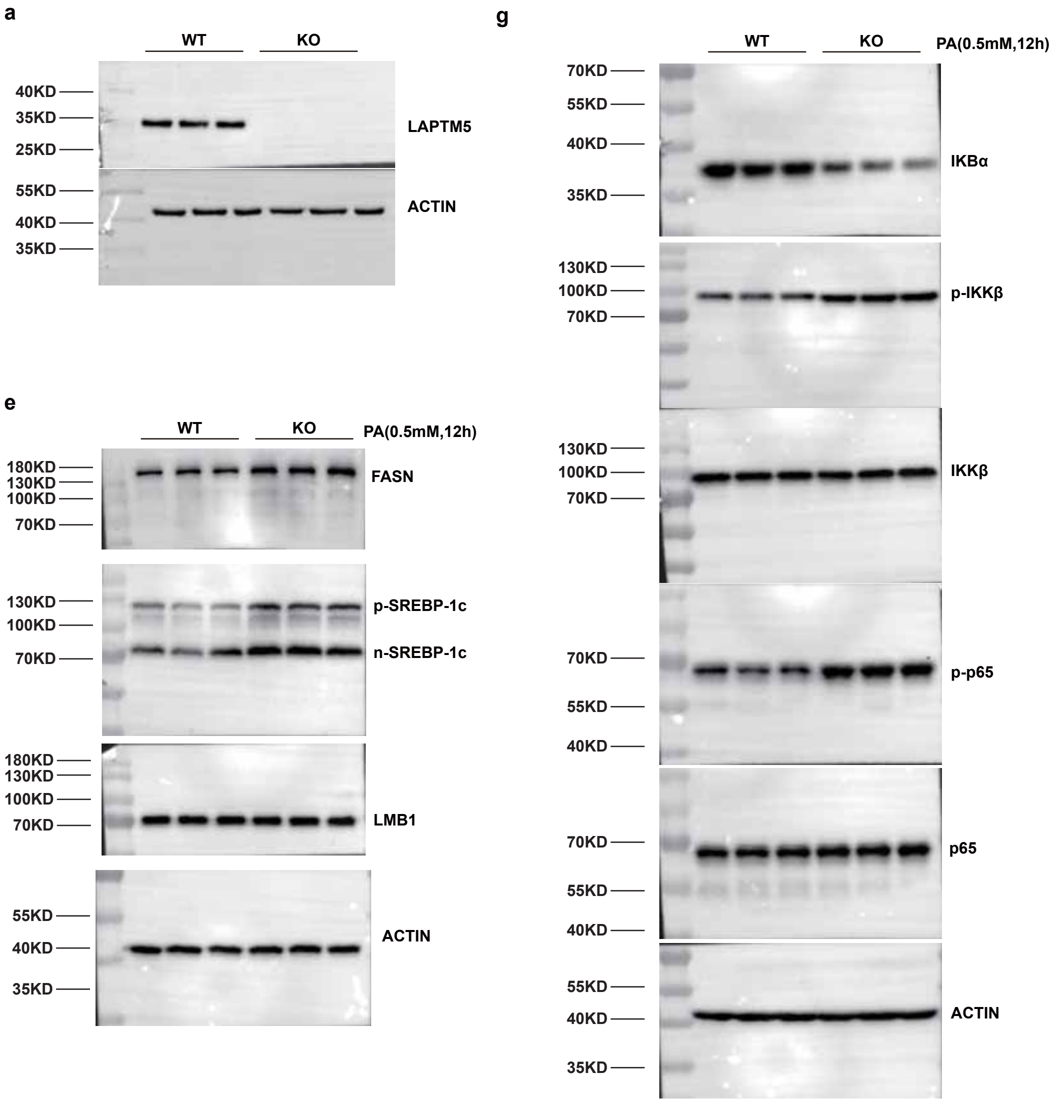
a
WT
KO
40KD
35KD
25KD
LAPTM5
55KD
40KD
35KD
ACTIN
e
WT
KO
PA(0.5mM,12h)
180KD
130KD
100KD
70KD
FASN
130KD
100KD
70KD
p-SREBP-1c
n-SREBP-1c
180KD
130KD
100KD
70KD
LMB1
55KD
40KD
35KD
ACTIN
g
WT
KO
PA(0.5mM,12h)
70KD
55KD
40KD
35KD
IKBα
130KD
100KD
70KD
p-IKKβ
130KD
100KD
70KD
IKKβ
70KD
55KD
40KD
p-p65
70KD
55KD
40KD
p65
55KD
40KD
35KD
ACTIN

figure 4

a

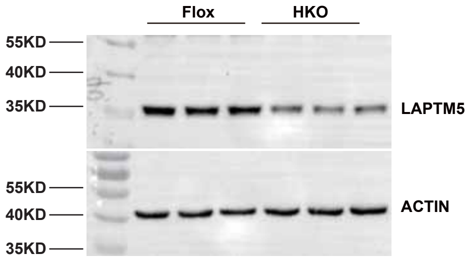

Flox
HKO
55KD
40KD
35KD
LAPTM5
55KD
40KD
35KD
ACTIN

l

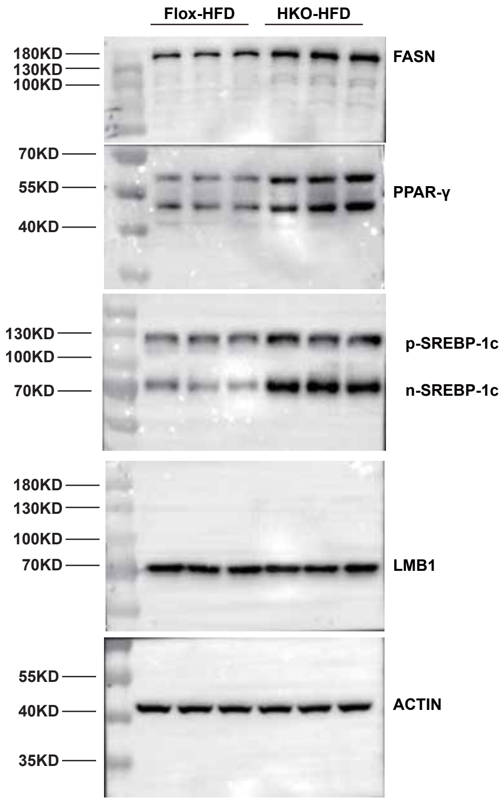

Flox-HFD
HKO-HFD
180KD
130KD
100KD
FASN
70KD
55KD
40KD
PPAR-γ
130KD
100KD
70KD
p-SREBP-1c
n-SREBP-1c
180KD
130KD
100KD
70KD
LMB1
55KD
40KD
35KD
ACTIN

figure 6

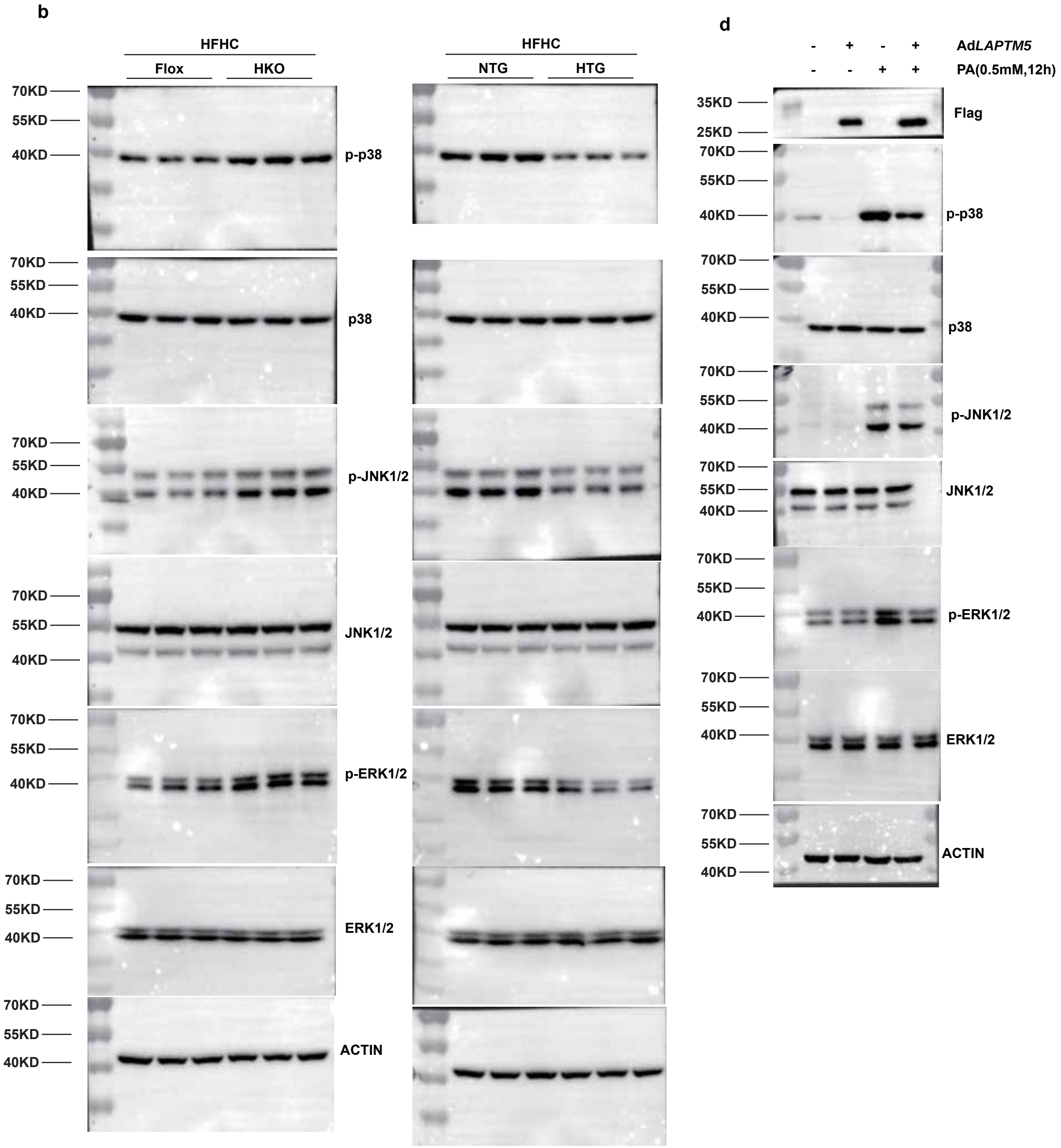
b
HFHC
Flox
HKO
HFHC
NTG
HTG
70KD
55KD
40KD
p-p38
p38
p-JNK1/2
JNK1/2
p-ERK1/2
ERK1/2
ACTIN
d
AdLAPTM5
PA(0.5mM,12h)
35KD
25KD
Flag
p-p38
p38
p-JNK1/2
JNK1/2
p-ERK1/2
ERK1/2
ACTIN

e

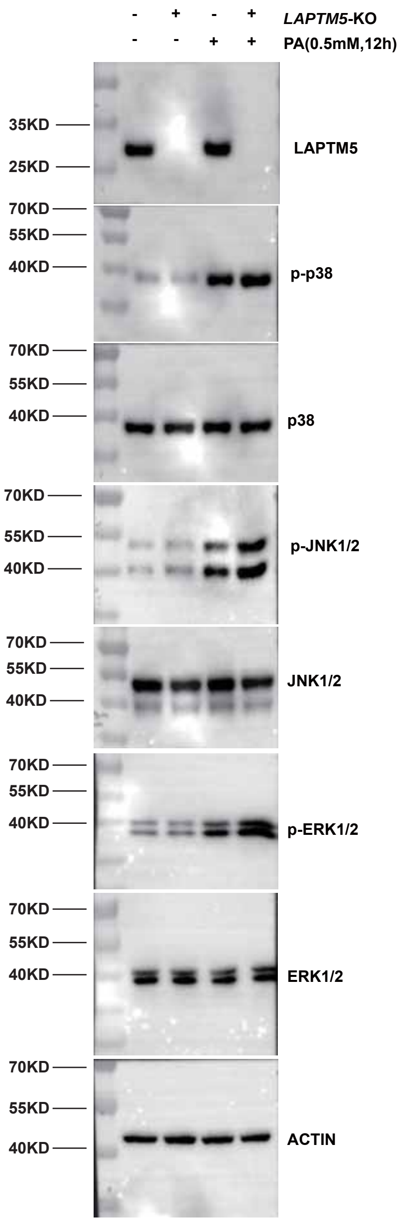
LAPTM5-KO
PA(0.5mM,12h)
35KD
25KD
LAPTM5
70KD
55KD
40KD
p-p38
70KD
55KD
40KD
p38
70KD
55KD
40KD
p-JNK1/2
70KD
55KD
40KD
JNK1/2
70KD
55KD
40KD
p-ERK1/2
70KD
55KD
40KD
ERK1/2
70KD
55KD
40KD
ACTIN

g

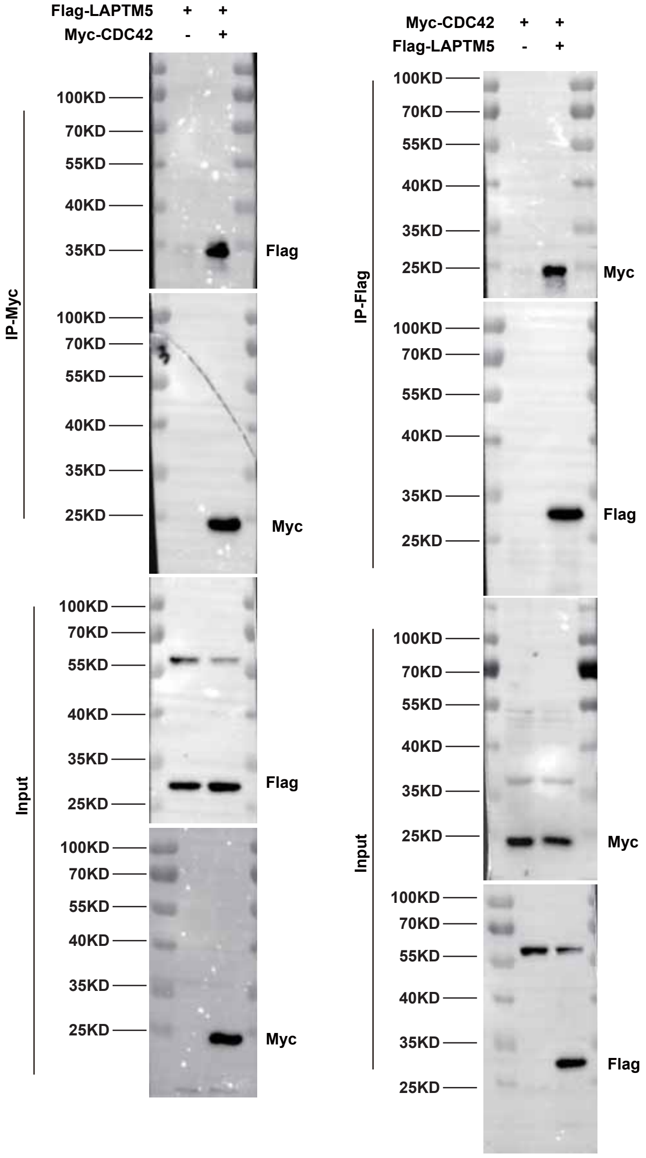
Flag-LAPTM5
Myc-CDC42
IP-Myc
100KD
70KD
55KD
40KD
35KD
Flag
100KD
70KD
55KD
40KD
35KD
25KD
Myc
Input
100KD
70KD
55KD
40KD
35KD
25KD
Flag
100KD
70KD
55KD
40KD
35KD
25KD
Myc
Myc-CDC42
Flag-LAPTM5
IP-Flag
100KD
70KD
55KD
40KD
35KD
25KD
Myc
100KD
70KD
55KD
40KD
35KD
25KD
Flag
Input
100KD
70KD
55KD
40KD
35KD
25KD
Myc
100KD
70KD
55KD
40KD
35KD
25KD
Flag

h

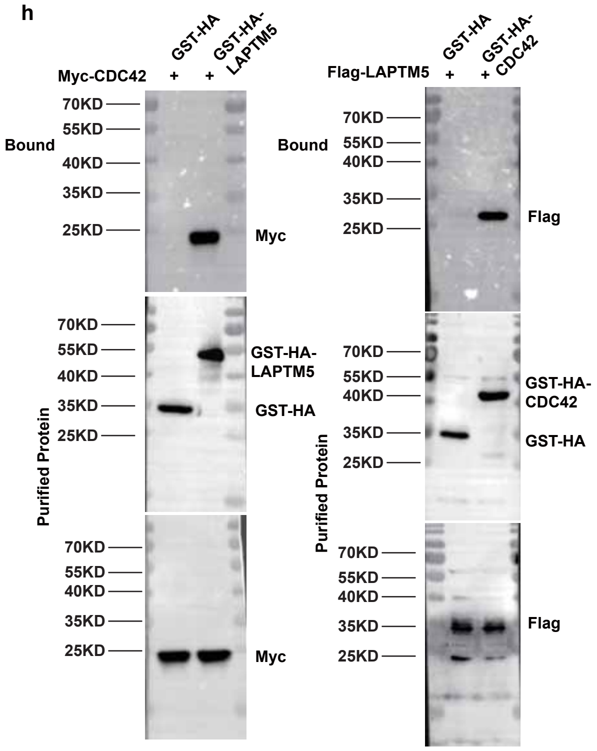
GST-HA
GST-HA-LAPTM5
Myc-CDC42
Bound
70KD
55KD
40KD
35KD
25KD
Myc
Purified Protein
70KD
55KD
40KD
35KD
25KD
GST-HA-LAPTM5
GST-HA
70KD
55KD
40KD
35KD
25KD
Myc
GST-HA
GST-HA-CDC42
Flag-LAPTM5
Bound
70KD
55KD
40KD
35KD
25KD
Flag
Purified Protein
70KD
55KD
40KD
35KD
25KD
GST-HA-CDC42
GST-HA
70KD
55KD
40KD
35KD
25KD
Flag

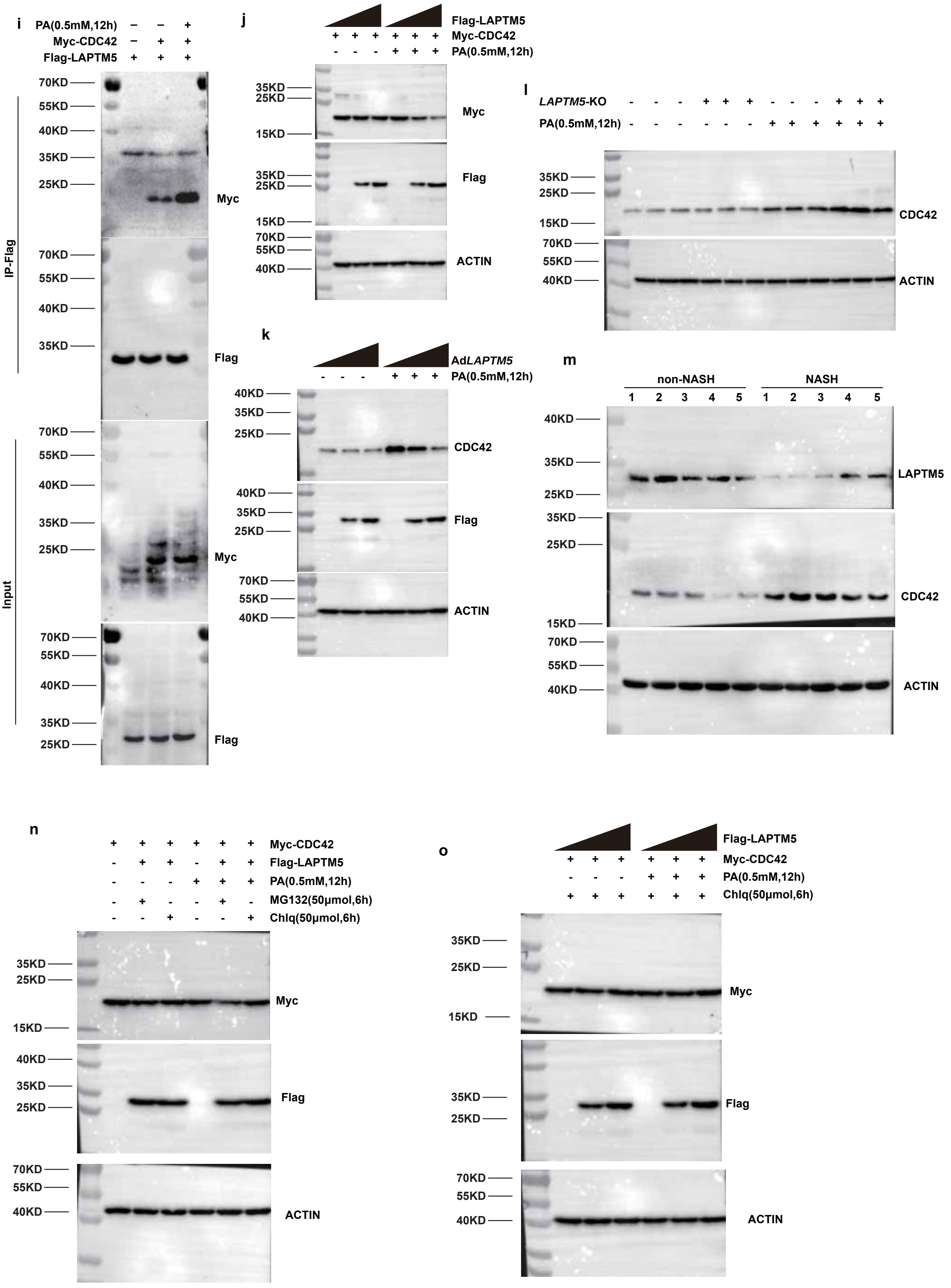
i
PA(0.5mM,12h) – – +
Myc-CDC42 – + +
Flag-LAPTM5 + + +
IP-Flag
Input
70KD
55KD
40KD
35KD
25KD
Myc
Flag
j
Flag-LAPTM5
Myc-CDC42 + + + + + +
PA(0.5mM,12h) - - - + + +
35KD
25KD
15KD
70KD
55KD
40KD
Myc
Flag
ACTIN
k
AdLAPTM5
PA(0.5mM,12h) - - - + + +
40KD
35KD
25KD
70KD
55KD
CDC42
Flag
ACTIN
l
LAPTM5-KO - - - + + + - - - + + +
PA(0.5mM,12h) - - - - - - + + + + + +
35KD
25KD
15KD
70KD
55KD
40KD
CDC42
ACTIN
m
non-NASH
NASH
1 2 3 4 5 1 2 3 4 5
40KD
35KD
25KD
15KD
70KD
55KD
LAPTM5
CDC42
ACTIN
n
Myc-CDC42 + + + + + +
Flag-LAPTM5 - + + - + +
PA(0.5mM,12h) - - - + + +
MG132(50μmol,6h) - + - - + -
Chlq(50μmol,6h) - - + - - +
35KD
25KD
15KD
40KD
70KD
55KD
Myc
Flag
ACTIN
o
Flag-LAPTM5
Myc-CDC42 + + + + + +
PA(0.5mM,12h) - - - + + +
Chlq(50μmol,6h) + + + + + +
35KD
25KD
15KD
70KD
55KD
40KD
Myc
Flag
ACTIN

figure 7

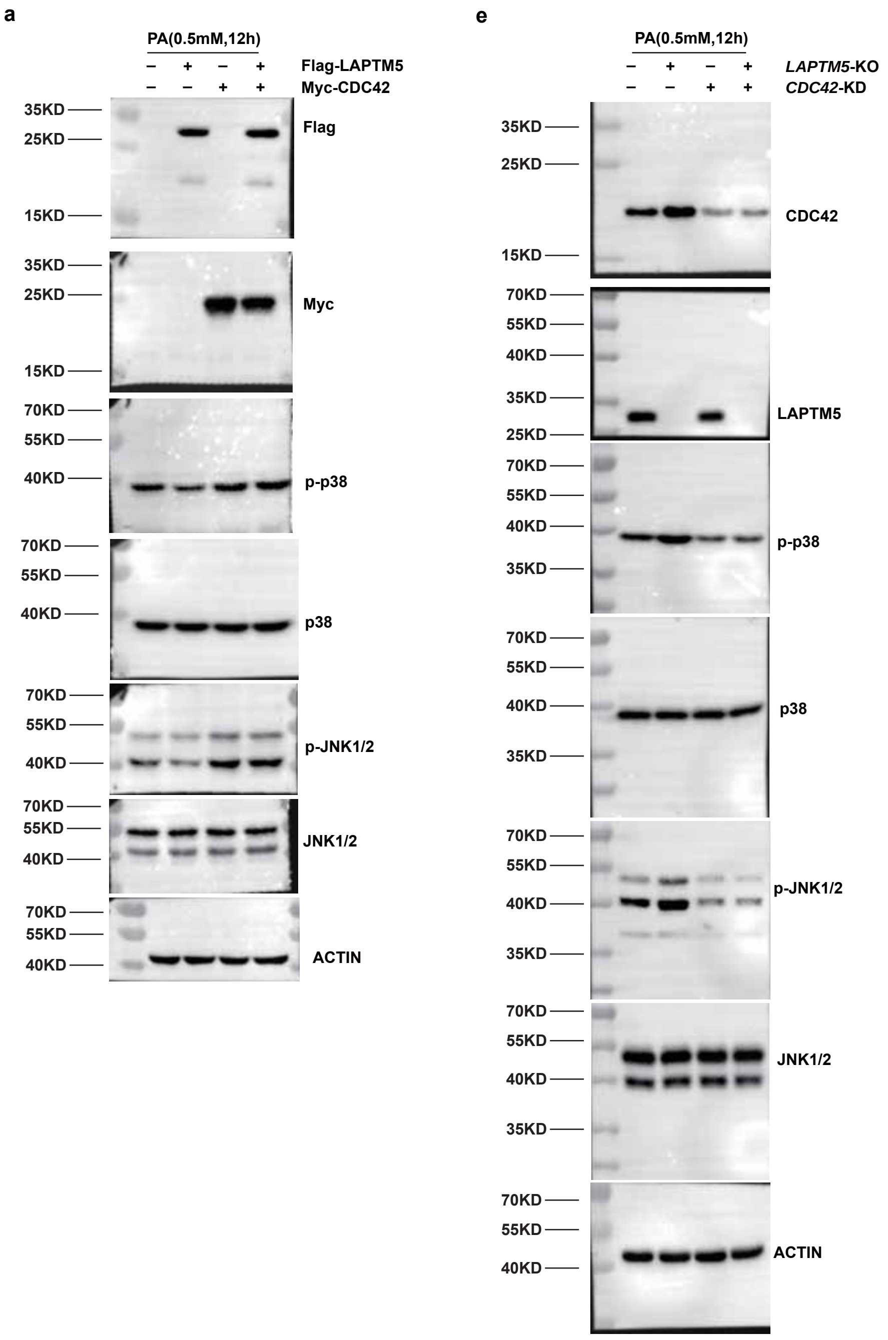

a
PA(0.5mM,12h)
Flag-LAPTM5
Myc-CDC42
35KD
25KD
15KD
Flag
Myc
70KD
55KD
40KD
p-p38
p38
p-JNK1/2
JNK1/2
ACTIN
e
LAPTM5-KO
CDC42-KD
CDC42
LAPTM5

figure 8

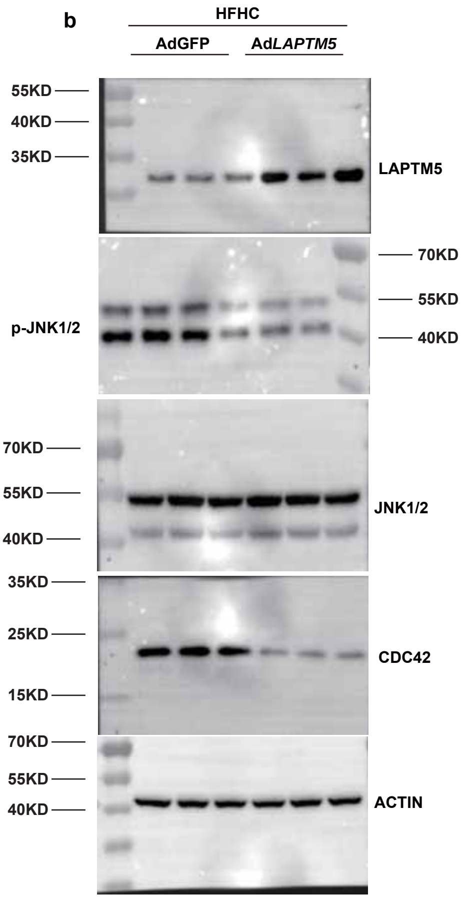
b
HFHC
AdGFP
AdLAPTM5
55KD
40KD
35KD
LAPTM5
70KD
55KD
40KD
p-JNK1/2
70KD
55KD
40KD
JNK1/2
35KD
25KD
15KD
CDC42
70KD
55KD
40KD
ACTIN

figure S1

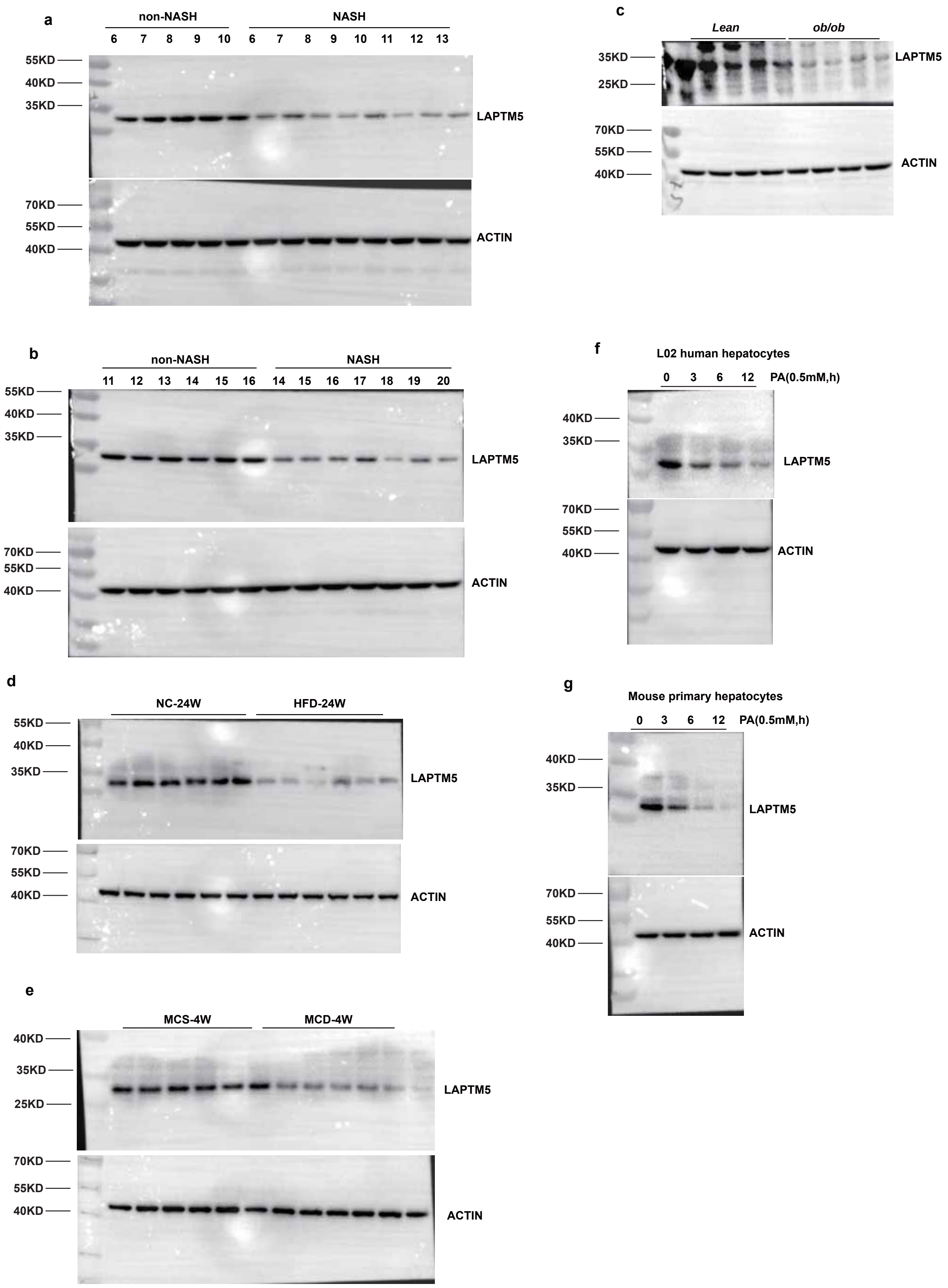
a
non-NASH
NASH
6 7 8 9 10 6 7 8 9 10 11 12 13
55KD
40KD
35KD
LAPTM5
70KD
55KD
40KD
ACTIN
b
non-NASH
NASH
11 12 13 14 15 16 14 15 16 17 18 19 20
55KD
40KD
35KD
LAPTM5
70KD
55KD
40KD
ACTIN
c
Lean
ob/ob
35KD
25KD
LAPTM5
70KD
55KD
40KD
ACTIN
d
NC-24W
HFD-24W
55KD
40KD
35KD
LAPTM5
70KD
55KD
40KD
ACTIN
e
MCS-4W
MCD-4W
40KD
35KD
25KD
LAPTM5
70KD
55KD
40KD
ACTIN
f
L02 human hepatocytes
0 3 6 12 PA(0.5mM,h)
40KD
35KD
LAPTM5
70KD
55KD
40KD
ACTIN
g
Mouse primary hepatocytes
0 3 6 12 PA(0.5mM,h)
40KD
35KD
LAPTM5
70KD
55KD
40KD
ACTIN

figure S2

a

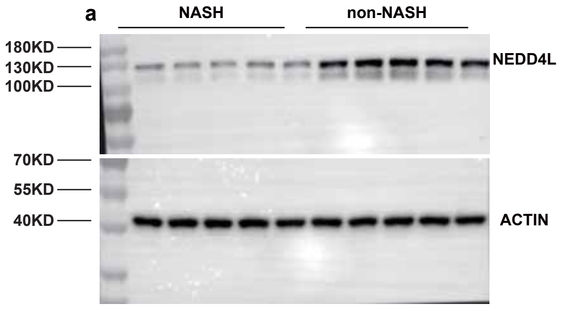

b

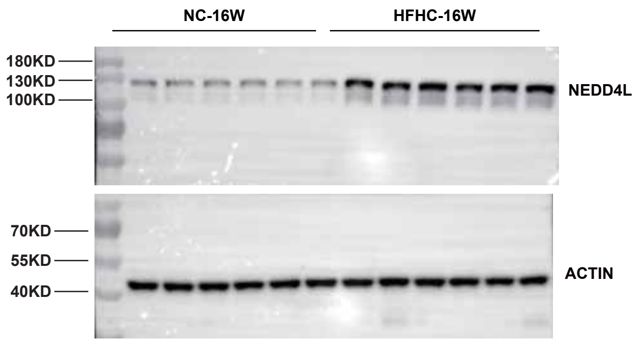

c

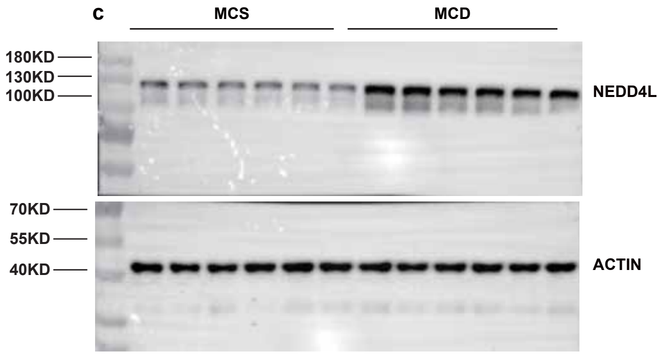

d

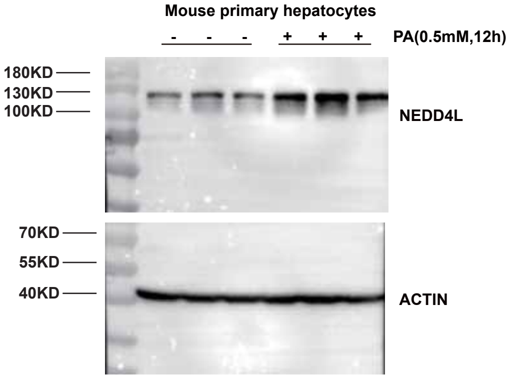

e

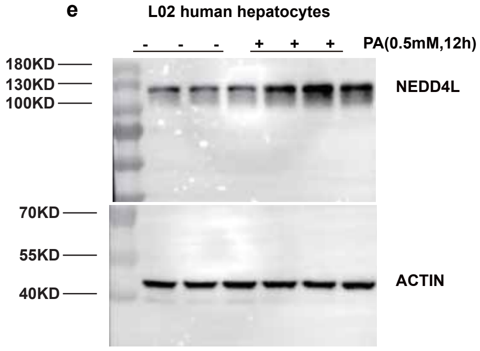

figure S3

e

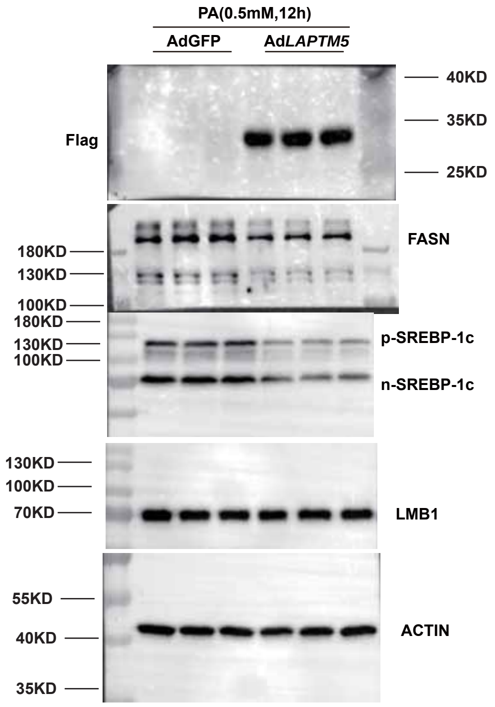

figure S3

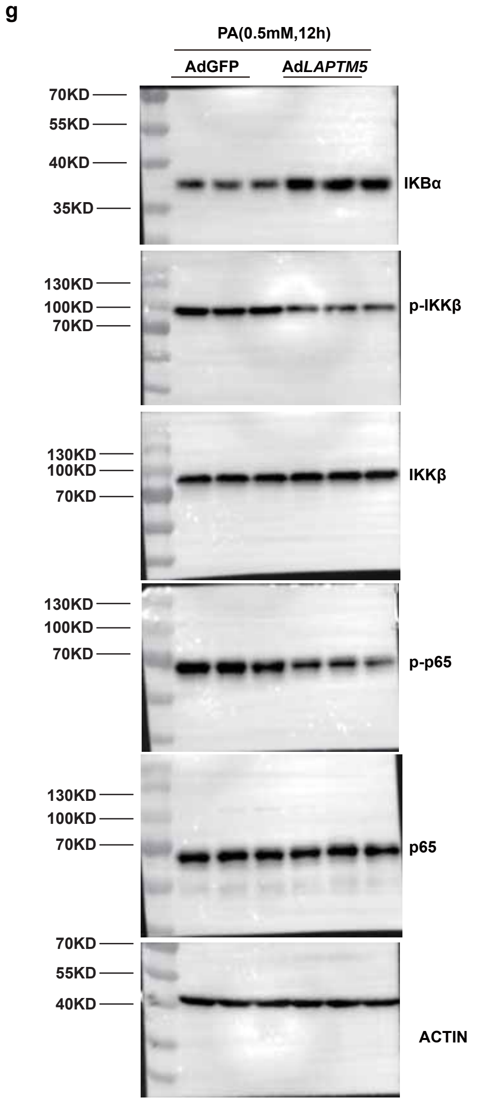

figure S8

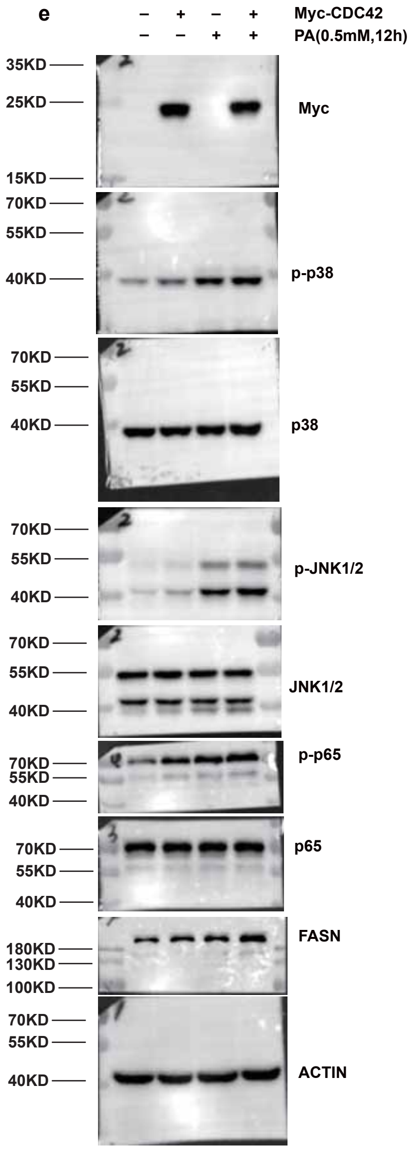

figure S7

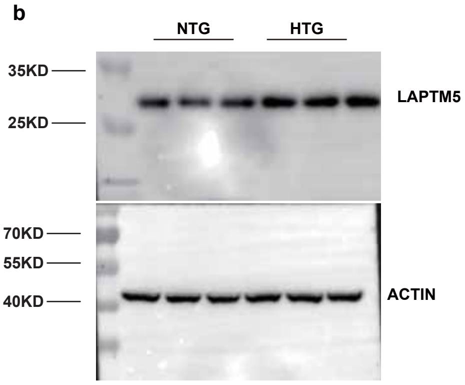

Supplement: Supplementary file 4 — Source Data [file 41467_2023_37908_MOESM4_ESM.zip › Source Data/Source Data.pdf]
